# Harnessing Native-Resolution 2D Embeddings for Lung Cancer Classification: A Feasibility Study with the RAD-DINO Self-supervised Foundation Model

**DOI:** 10.1007/s10278-025-01748-4

**Published:** 2025-12-19

**Authors:** Md.Enamul Hoq, Lawrence Tarbox, Donald Johann, Linda Larson-Prior, Fred Prior

**Affiliations:** 1 Department of Biomedical Informatics, University of Arkansas for Medical Sciences, AR Little Rock, USA; 2 Department of Neuroscience, University of Arkansas for Medical Sciences, Little Rock, AR USA

**Keywords:** Lung cancer screening, Low-dose computed tomography (LDCT), Deep learning, Self-supervised learning, RAD-DINO, National Lung Screening Trial (NLST)

## Abstract

Low‑dose CT (LDCT) screening reduces lung cancer mortality but yields high false‑positive findings, motivating practical AI that aligns with slice‑based clinical review. We evaluate a label‑efficient 2D pipeline that uses frozen RAD‑DINO embeddings from native‑resolution axial slices and a lightweight multilayer perceptron for patient‑level risk estimation via mean aggregation and isotonic calibration. Using the NLST CT arm with outcomes defined over a 0–24‑month window, we construct a fixed patient‑level split (one CT per patient; no cross‑split leakage) and perform 25 repeated imbalanced test draws (~ 6% prevalence) to approximate screening conditions. At screening prevalence, RAD‑DINO + MLP achieves PR‑AUC = 0.705 (calibrated; raw 0.554) and ROC‑AUC = 0.817 (raw; 0.736 calibrated), with improved probability reliability following calibration; operating points are selected on validation and reported on test. For secondary ablations only, a near‑balanced cohort (*N* = 1984) yields accuracy 0.966, precision 0.974, recall 0.973, F1 0.973, and ROC‑AUC 0.912. Beyond classification, retrieval with triplet‑fine‑tuned embeddings attains Precision@5 = 0.853. Interpretability analyses show that cancer cases sustain higher top‑k slice scores and that directional SHAP concentrates on a small subset of high‑probability slices; label‑colored t‑SNE provides qualitative views of embedding structure. Limitations include single‑cohort evaluation, lack of Lung‑RADS labels in public NLST, and a CXR → CT pretraining shift; future work will pursue external validation and CT‑native self‑supervised continuation. Overall, frozen 2D foundation embeddings provide a strong, transparent, and computationally practical starting point for LDCT screening workflows under realistic prevalence.

## Introduction

### Background

Lung cancer remains the leading cause of cancer-related death worldwide. Early detection through low-dose computed tomography (LDCT) screening has been shown to significantly reduce mortality [1]. In the National Lung Screening Trial (NLST), annual LDCT screening in high-risk patients yielded a ~ 20% reduction in lung cancer mortality compared to chest radiography [2–4]. A subsequent European trial (NELSON) reported a ~ 24% reduction in lung cancer mortality at 10-year follow-up with volumetric LDCT screening [5]. These landmark studies led to LDCT being adopted in clinical screening guidelines. However, LDCT screening also produces a high rate of false positives and negatives—on the order of 95% or more of positive screens do not actually harbor cancer [1]. This high false-positive rate burdens patients with unnecessary follow-up tests and highlights the need for improved methods to distinguish malignant from benign findings in screening.

Recent advances in artificial intelligence have shown great promise in assisting radiologists with LDCT interpretation [6]. In particular, deep learning models can be trained to detect lung cancer on CT scans. Recent efforts in 3D CT foundation modeling have demonstrated the potential of volumetric pretraining for thoracic disease detection. Notably, McConnell et al. proposed an open-source, computationally frugal 3D foundation model for lung cancer screening [7], and Niu et al. introduced a multimodal, multitask 3D foundation model for lung cancer screening [8]. Our work complements these approaches by investigating whether a lightweight 2D self-supervised representation, trained on large-scale chest X-rays, can achieve comparable clinical feasibility with reduced computational and data demands. Among the earliest demonstrations of deep learning in this domain, Causey et al. presented a pioneering 3D convolutional neural network (CNN) that predicted lung nodule malignancy directly from LDCT volumes where a 3D CNN was trained for malignancy prediction on LIDC-IDRI nodules (AUC ≈0.95), establishing a precedent for volumetric deep learning in thoracic imaging [6]. This work established a key precedent for volumetric deep learning in thoracic imaging and highlighted the feasibility of end-to-end AI for malignancy classification [9]. Based on this foundation, an example by Ardila et al. employed a 3D convolutional neural network (CNN) to predict lung cancer risk from full LDCT volumes, achieving a state-of-the-art area under the ROC curve (AUC) of 94.4% on 6716 NLST cases and even outperforming expert radiologists in certain settings considering the different time points from the patients’ visiting records [9].

These successes illustrate the growing reliance on full volumetric analysis in lung cancer AI pipelines. Volumetric models naturally leverage the entire scan and utilize cross-slice continuity—such as recognizing nodules that persist across consecutive slices. Indeed, 3D CNNs typically achieve higher specificity than 2D slice-based models, potentially reducing false positives through volumetric context. For instance, Yu et al. found that a 3D CNN substantially reduced false-positive nodule detections compared to a 2D CNN in a lung screening task (achieving AUC 0.954 vs. 0.931), albeit with greater model complexity and computation requirements [10].

Despite these advantages, the volumetric paradigm presents practical challenges. In practice, radiologists still review CT scans as a stack of 2D images (axial slices) rather than manipulating a 3D volume. Furthermore, 3D models require preprocessing steps that often introduce interpolation artifacts, especially when reconstructing isotropic volumes from anisotropic CT slices [11, 12]. This process, while necessary for 3D network inputs, can obscure subtle imaging features critical for early cancer detection. To estimate voxel intensities between discrete axial slices, interpolation is typically applied, as expressed in Eq. (1):

1 $$I\left(x,y,z\right)\approx\underset k{\sum\;\;}I\left(x,y,z_k\right).w_k\left(z\right),\;where\underset k{\sum\;}W_k\left(z\right)=1$$

Here, *I*(x,y,z_k_) represents known 2D axial slices, and *w*_k_(z) are the interpolation weights. While enabling volumetric consistency, this introduces approximations that may dilute diagnostically important signals. Moreover, 3D CNNs require significantly greater computational resources and have more parameters, increasing the risk of overfitting particularly when annotated data is limited [13]. In contrast, 2D models operate on each slice in its native resolution, avoiding interpolation, redcing model complexity, and offering improved interpretability by pinpointing predictions to an exact slice.

This study, therefore, pivots from the prevalent volumetric paradigm to explore the feasibility of a 2D slice-based alternative. We investigate a state-of-the-art 2D self-supervised foundation model, RAD-DINO, which was pretrained on a large corpus of X-ray medical images [14]. To comprehensively evaluate the utility of these 2D embeddings, we assess them on two distinct downstream tasks: (1) a high-fidelity classification task using the raw embeddings to test their out-of-the-box discriminative power and (2) a patient-level retrieval task using embeddings fine-tuned for semantic similarity to test their adaptability. This dual analysis demonstrates the potential of the RAD-DINO foundation model as a flexible starting point for various clinical AI applications. Our aim is to establish a strong baseline for 2D self-supervised approaches, laying the groundwork for future validation in clinically representative settings. For readers interested in a contextual reference, a similar analysis using the volumetric CT-Foundation model on an imbalanced NLST cohort is provided in the Supplementary Materials [15].

## Methods

### Data and Preprocessing

#### Dataset

We utilized the publicly available LDCT scans and outcomes from the National Lung Screening Trial (NLST) for training and evaluation [2, 3]. NLST [16] was a multi-center randomized trial that enrolled over 50,000 high-risk individuals (aged 55–74 with a heavy smoking history) to undergo annual screenings with LDCT versus chest X-ray [2]. In our study, we focus on the CT arm of NLST. The imaging data (DICOM format) and accompanying clinical outcomes were obtained via the National Cancer Institute’s The Cancer Imaging Archive (TCIA) [3].

Each CT examination was assigned a patient-level outcome label (“cancer” or “non-cancer”). A case was labeled cancer if the patient received a lung cancer diagnosis within 24 months of the index screening LDCT (biopsy-confirmed or clinically adjudicated), consistent with NLST follow-up practice and prior NLST-derived analyses [1]. Scans were labeled non-cancer if no lung cancer diagnosis occurred during that 0–24-month interval. This outcome-based scheme prioritizes clinically consequential events; we note that some images labeled “cancer” may not display a radiographically obvious tumor at baseline (e.g., occult or extremely subtle lesions). To assess robustness, we will perform sensitivity analyses using alternate windows (12 and 36 months). We included one scan per patient (earliest available screening round—baseline unless stated) and enforced patient-level train/validation/test splits to prevent slice- or subject-level leakage.

#### Cohort Selection and Fixed Patient-Level Split

We formed a single patient-level split with no overlap: Train = 1031 (541 pos/490 neg), Val = 258 (126/132), Test = 695 (355/340). From the fixed Test pool, we constructed 25 independent draws targeting ≈6% cancer prevalence by keeping *all* negatives and randomly sub-sampling positives (without replacement) to the target prevalence; Train/Val are unchanged. Slice scores are aggregated to the patient level by mean (primary). An isotonic calibrator is fit on Val and applied to Test. We report PR-AUC (primary), ROC-AUC, Brier, and ECE@10; operating points (Max-F1 and sensitivity targets) are selected on Val and evaluated on Test. Results are summarized by median and 95% percentile interval across repeats. Train, Val, and Test patient counts for the fixed split are given in Table 1.

#### Balanced Development Cohort (Secondary)

Balanced Development Cohort (Secondary) For ablations only, we used a near-balanced cohort *N* = 1984 (1022 cancer, 962 non-cancer) with stratified 80/20 splits. Counts represent unique patients (one CT per patient); train/validation/test partitions were enforced at the patient level to preclude cross-split slice leakage. We do not base clinical claims on these numbers.

#### Image Processing

Each LDCT scan was originally acquired as a series of 2D axial slices (typically 1–3 mm thickness) stored in DICOM. For the 2D RAD-DINO pipeline, minimal preprocessing was done—we used each axial slice image at its native resolution. We applied standard intensity windowing to focus on lung tissue (e.g., Hounsfield unit window ~ [− 1000, 400] HU), and then normalized pixel values per slice. No global resizing was applied, so each slice preserved the original pixel spacing (on the order of 0.5–0.8 mm in-plane). The 2D slices were then processed independently through the model.

**Table 1:** Patient counts by split (positives/negatives/total). Counts are unique patients; one CT per patient; patient-level split with no cross-split slice leakage

Split	Positives	Negatives	Total
Training	541	490	1031
Validation	126	132	258
Test	355	340	695

### Model Pipeline and Feature Analysis

#### RAD-DINO Model and Patient-Level Prediction

We employed RAD-DINO, a deep neural network pretrained using chest X-ray images, specifically designed for processing individual two-dimensional (2D) slices. The architecture of RAD-DINO is based on a Vision Transformer (ViT) [17] backbone initialized using self-supervised learning via the DINO algorithm (Distillation with No Labels) [18]. RAD-DINO’s pretraining on extensive unlabeled chest X-ray datasets and self-supervised contrastive learning capability via the DINOv2 backbone enabled it to effectively learn generalizable anatomical and pathological features relevant to thoracic imaging [18].

Our primary evaluation uses a clinically realistic screening prevalence. We first define a fixed, patient-level split with no leakage—one CT per patient (baseline round unless otherwise noted)—yielding Train = 1031 (541 pos/490 neg), Val = 258 (126/132), Test = 695 (355/340). To approximate real-world prevalence, we create repeated imbalanced test *draws* from this fixed split by keeping all test negatives and randomly subsampling positives to ≈6% prevalence; we run 25 repeats and summarize patient-level performance with medians and 95% percentile intervals. For completeness and ablations, we also analyze a class-balanced NLST subset (≈1:1 cancer: non-cancer, 1984 patients) constructed by including all available cancers and randomly sampling a matched number of non-cancers; this balanced subset is used only for secondary analyses (e.g., visualizations/interpretability), not to claim prevalence-realistic performance.

We use RAD-DINO *as-is* (no CT fine-tuning) to encode each axial slice into a 768-D embedding (≈1.3 M slices total across cohorts). A lightweight MLP head is trained on these frozen slice embeddings to predict slice-level malignancy scores; per-patient scores are obtained by mean aggregation across slices. The MLP is optimized with Adam with standard normalization/augmentation. Isotonic calibration is fit only on the validation set and applied to test predictions; operating thresholds (Max-F1 and sensitivity-targeted) are selected on validation and evaluated on test. We include two 2D baselines under identical preprocessing, aggregation, splits, and calibration: (i) ResNet-18 (ImageNet-pretrained) trained slice-wise with patient-level mean aggregation [19]; (ii) a linear probe (logistic regression) on frozen RAD-DINO slice embeddings. All metrics are reported at the patient level using the same validation-trained calibrator.

#### Patient-Level Prediction

For inference, RAD-DINO embeddings were computed for all axial slices of a patient’s CT scan. Slice-level probability scores generated by the classifier were aggregated to produce a patient-level cancer prediction. Multiple aggregation strategies were explored, including the maximum score (max-pooling), mean score, and logistic regression on slice-level statistics. The mean aggregation approach yielded the highest patient-level predictive accuracy and F1-score, aligning closely with clinical reasoning—where a single malignant slice is sufficient for diagnosing lung cancer. For each patient with N slices, slice probabilities $\widehat{{p}_{i}}$ are aggregated by the mean. Thus, our final reported patient-level cancer probability is defined as in Eq. (2):

2 $$\widehat Ppatient\;=\frac1N\;\overset N{\underset{i\mathit-\mathit1}{\mathit\sum}}\widehat Pi$$

where $\widehat{{p}_{i}}$ is the slice-level probability for slice *i*. The threshold for classification was tuned based on validation data to optimize the F1-score.

#### Fine-Tuning and Evaluation for a Retrieval Task

To assess the adaptability of the embeddings for a semantic similarity task, we implemented a fine-tuning procedure. The initial 768-dimensional patient embeddings (mean-pooled from slices) were projected into a 128-dimensional space by an encoder network (a two-layer MLP). This network was trained for 1000 epochs using a triplet margin loss function (margin = 1.0) [20]. This process explicitly optimizes the embedding space so that patients with the same diagnosis are closer to each other than to patients with a different diagnosis.

Retrieval performance of these fine-tuned embeddings was quantified using Precision at 5 (P@5). For each patient, we identified the top 5 most similar patients using cosine similarity and calculated the proportion that had the same label.

#### Statistical Validation and Interpretability

To validate the clinical relevance of the generated embeddings, we performed statistical analysis on the vector norms of the patient-level feature representations. An independent samples *T*-test and a Mann–Whitney *U* test were conducted to determine if there was a statistically significant difference between the embedding distributions of the cancer and non-cancer cohorts.

To provide insight into the model’s decision-making process, we employed SHAP (SHapley Additive exPlanations) [21]. This analysis was used to identify which of the 768 features in the RAD-DINO embedding vector had the most significant impact on the model’s output for predicting cancer. The embedding space was also visualized using dimensionality reduction techniques such as t-SNE to qualitatively assess class separability.

We computed slice-level attributions using SHAP on the model’s logit output. For each slice, we obtained directional *SHAP* values (positive values push toward “cancer,” negative values toward “non-cancer”). To summarize which RAD-DINO embedding dimensions drive decisions, we report (i) a beeswarm plot of the top-20 embedding features ranked by mean absolute SHAP and (ii) a bar chart of mean |SHAP| across the evaluation set. To test whether the model’s explanations concentrate where it “looks,” we sampled 60 patients (30 cancer/30 non-cancer) and, per patient, selected the top-5 probability slices (300 slices total). We compared *within-patient* directional SHAP for the top-2 slices vs. the remaining three using a paired Wilcoxon signed-rank test; we also report per-patient Spearman *ρ*between slice rank and directional SHAP. (Full plotting and statistics are in the Appendix.)

**Fig. 1:**
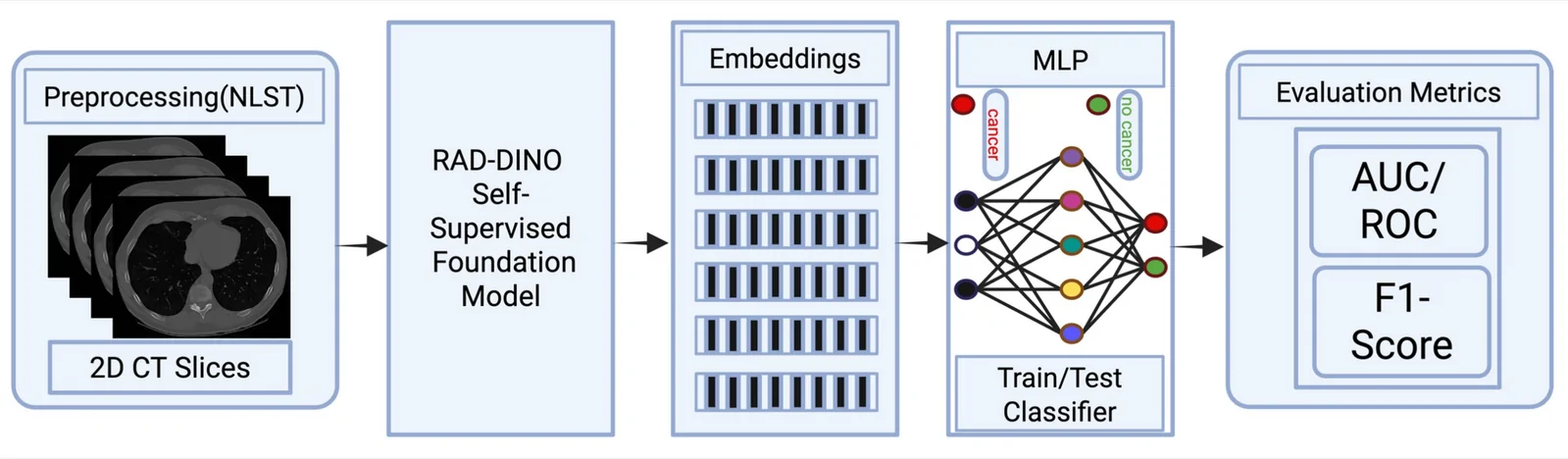
Overview of the lung cancer classification pipeline using RAD-DINO. Raw 2D CT slices from the NLST dataset are processed by the RAD-DINO model to generate high-dimensional embeddings. These embeddings are then used to train a classifier, and the results are evaluated to produce patient-level performance scores

Figure 1 illustrates the overall pipeline of our RAD-DINO-based classification framework for lung CT images. The process begins with preprocessing axial 2D CT slices from the NLST dataset. These slices are then passed through the RAD-DINO self-supervised foundation model to generate robust feature embeddings. The resulting embeddings serve as input to a lightweight multilayer perceptron (MLP) classifier, which is trained to distinguish between cancer and non-cancer cases. Finally, the classifier’s performance is evaluated using standard metrics such as AUC/ROC and F1-score, enabling quantitative assessment of model effectiveness.

## Results

### Classification Performance

**Fig. 2:**
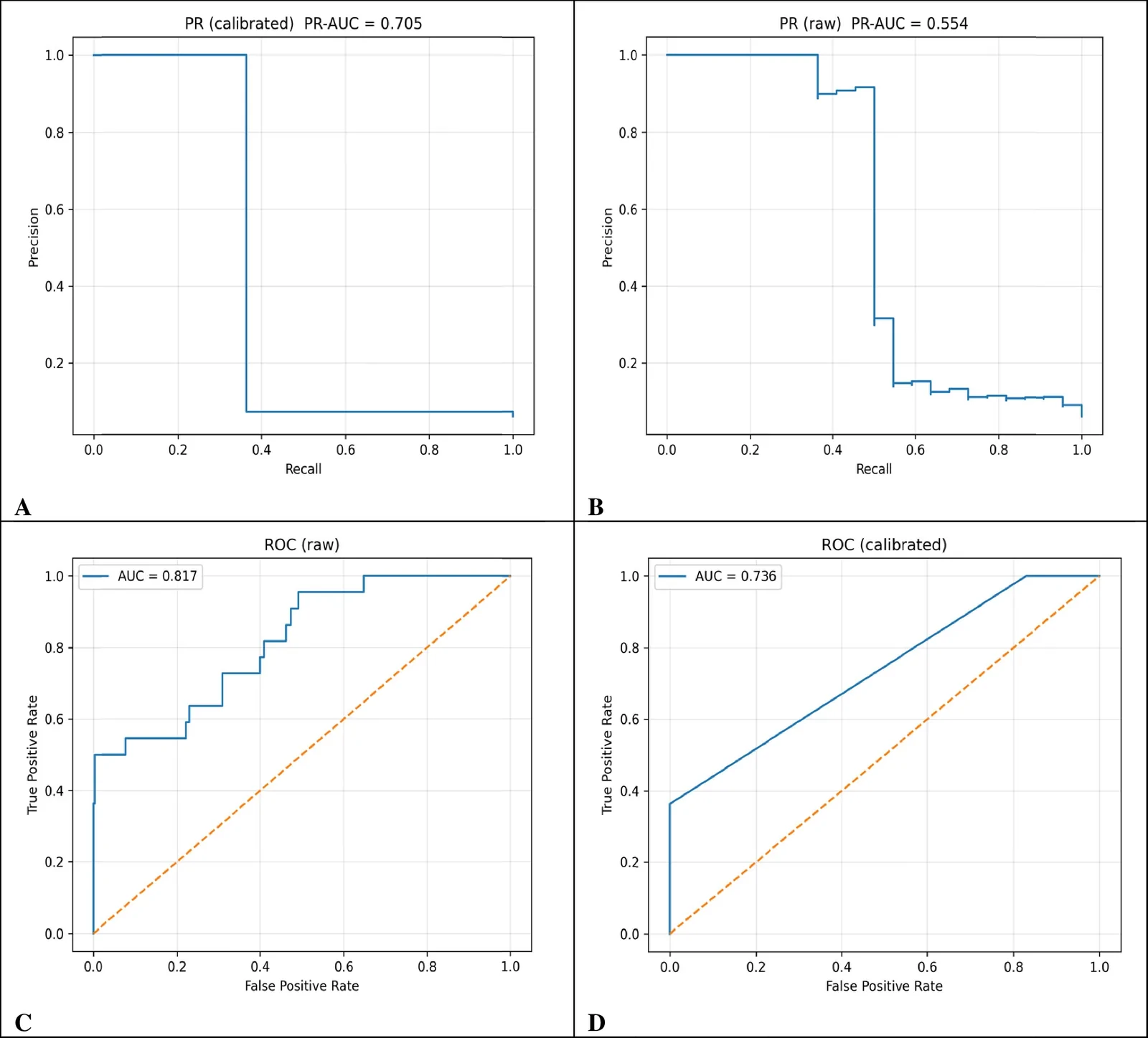
Discrimination under screening prevalence. **A** Precision–recall (calibrated) with PR-AUC ≈0.71. **B** Precision–recall (raw) with PR-AUC ≈0.55. **C** ROC (raw) with AUC ≈0.82. **D** ROC (calibrated) with AUC ≈0.74. Calibration improves precision across a wide recall range, as expected in a heavily imbalanced setting

On the fixed patient split with repeated imbalanced test draws (~ 6% cancer), a patient-level MLP trained on frozen RAD-DINO slice embeddings achieved PR-AUC 0.705 (calibrated; raw 0.554) and ROC-AUC 0.817 (raw; calibrated 0.736). Isotonic calibration substantially improved probability reliability (reliability curve near identity), and validation-selected operating points are reported on the test set (Fig. 2). Secondary analysis on the almost balanced development cohort used for ablation/model selection showed that performance was high (Table 2 and Figs. 6 and 7)—as expected under class balance—and is reported for completeness in the Supplement; we do not base screening utility on these numbers.

**Table 2:** Patient-level classification performance of RAD-DINO (2D model)

Model	Test set	Accuracy	Precision	Recall	F1-score
RAD-DINO	Balanced(~1:1)	0.966	0.974	0.973	0.973

### Screening-Prevalence Evaluation

**Fig. 3:**
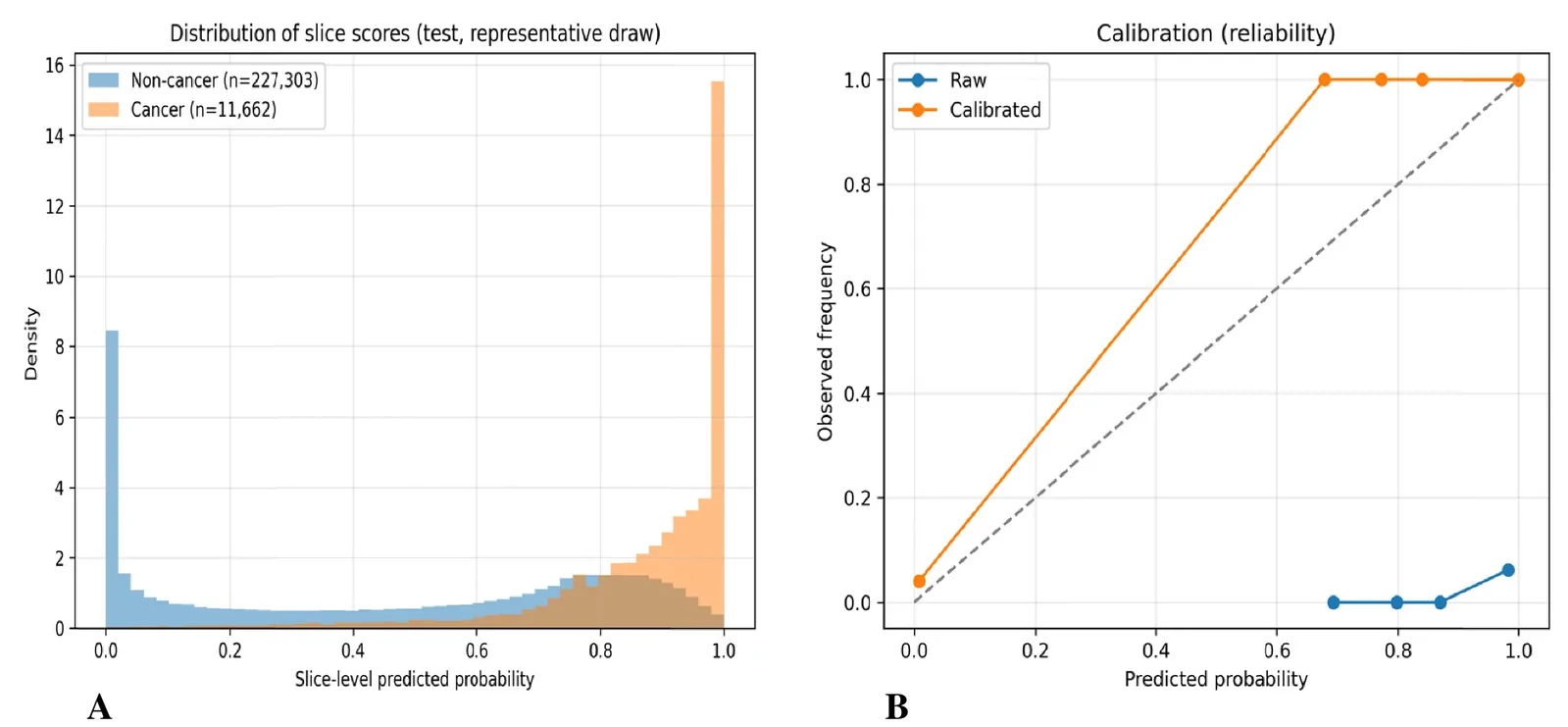
**A** Slice-level score distributions by class. Histogram (density) of slice-level predicted probabilities for test patients, separated by ground-truth class. Cancer slices are shifted to higher scores with a prominent high-confidence tail. **B** Probability calibration. Reliability diagram comparing raw vs. isotonic-calibrated probabilities on the test set (representative draw). Calibration brings the curve close to the y = x line, indicating well-calibrated risk estimates

**Fig. 4:**
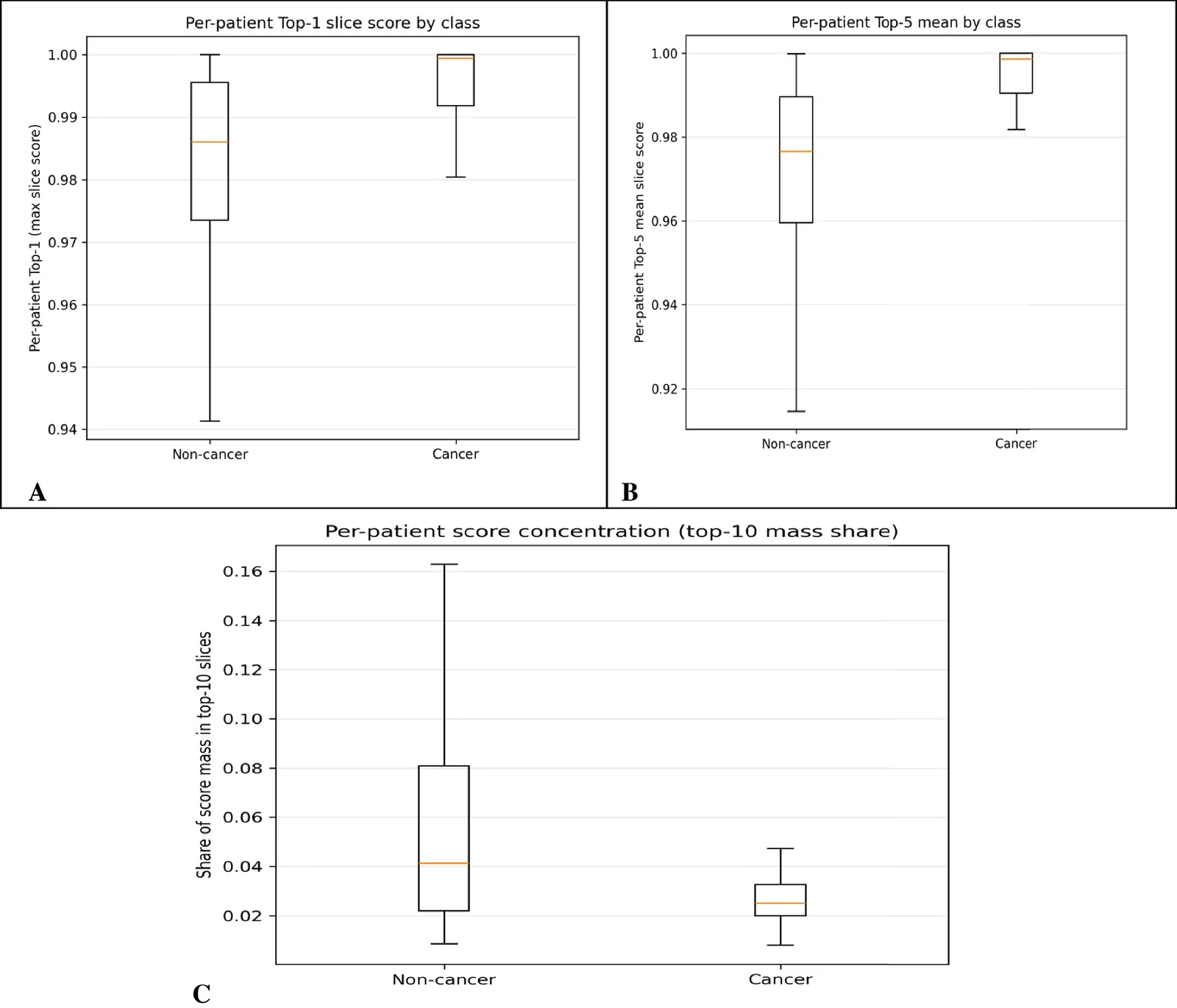
**A** Boxplots of the maximum slice score per patient for cancer vs. non-cancer in the test set (representative draw). Cancer patients show higher top-1 scores with tighter upper-quartile ranges. **B** Boxplots of the mean of the top-5 slice scores per patient. Cancer patients maintain elevated top-k confidence relative to non-cancer patients. **C** Share of total predicted probability mass contained in the top-10 slices per patient. Differences illustrate how a small slice subset concentrates model confidence

On a representative draw at ≈6% prevalence, the patient-level classifier trained on frozen RAD-DINO slice embeddings with a lightweight MLP head shows useful discrimination under class imbalance. The PR curve (Fig. 2A) yields a PR-AUC ≈0.71 after isotonic calibration (raw ≈0.55, Fig. 2B), reflecting improved precision at clinically relevant recall. ROC-AUC is ≈0.82 for raw scores (Fig. 2C) and ≈0.74 after calibration (Fig. 2D), a known effect when calibration slightly reorders a small set of cases yet improves probability quality. Reliability improves markedly after calibration, with the calibration curve closely tracking the identity line (Fig. 3A). Finally, slice-level score distributions show substantial class separation (Fig. 3B), and per-patient summaries confirm that cancer cases tend to sustain higher top-k slice scores (Fig. 4A–C); rank-vs-score traces illustrate how a handful of high-scoring slices dominate patient-level risk (see Appendix Figs. 18, 19, 20, and 21).

### Interpretability/Slice-Level Behavior

Beyond scalar metrics, we characterize how signal concentrates within volumes. Cancer patients exhibit consistently higher top-1 and top-5 slice scores (Fig. 4A–C) and a modestly different concentration pattern of probability mass across slices (Fig. 4C). Rank-score traces (Appendix Figs. 18, 19, 20, and 21) show that, for many positives, a small prefix of slices carries the majority of confidence—consistent with localized pathology—whereas negatives decline rapidly. These views support a clinically intuitive workflow: triage by patient score, then inspect the highest-scoring slices.

#### Balanced Development Cohort (Secondary Analysis)

The downstream classifier, trained on the pre-computed RAD-DINO embeddings, was evaluated on a held-out, balanced test set of 1984 patients. At the patient level, the model achieved an accuracy of 96.6%, a precision of 97.4%, a recall of 97.3%, and an F1-score of 97.3%. These strong performance metrics underscore the high quality of the features extracted by the RAD-DINO model without any task-specific fine-tuning. Table 2 summarizes these primary performance metrics.

**Fig. 5:**
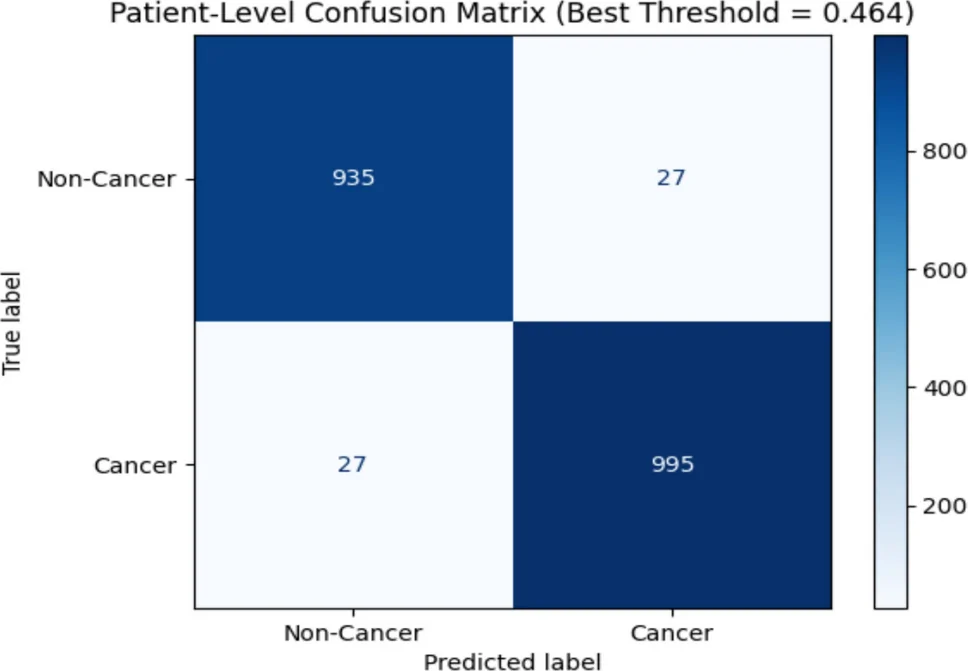
RAD-DINO patient-level confusion matrix on the balanced test set (threshold = 0.464)

**Fig. 6:**
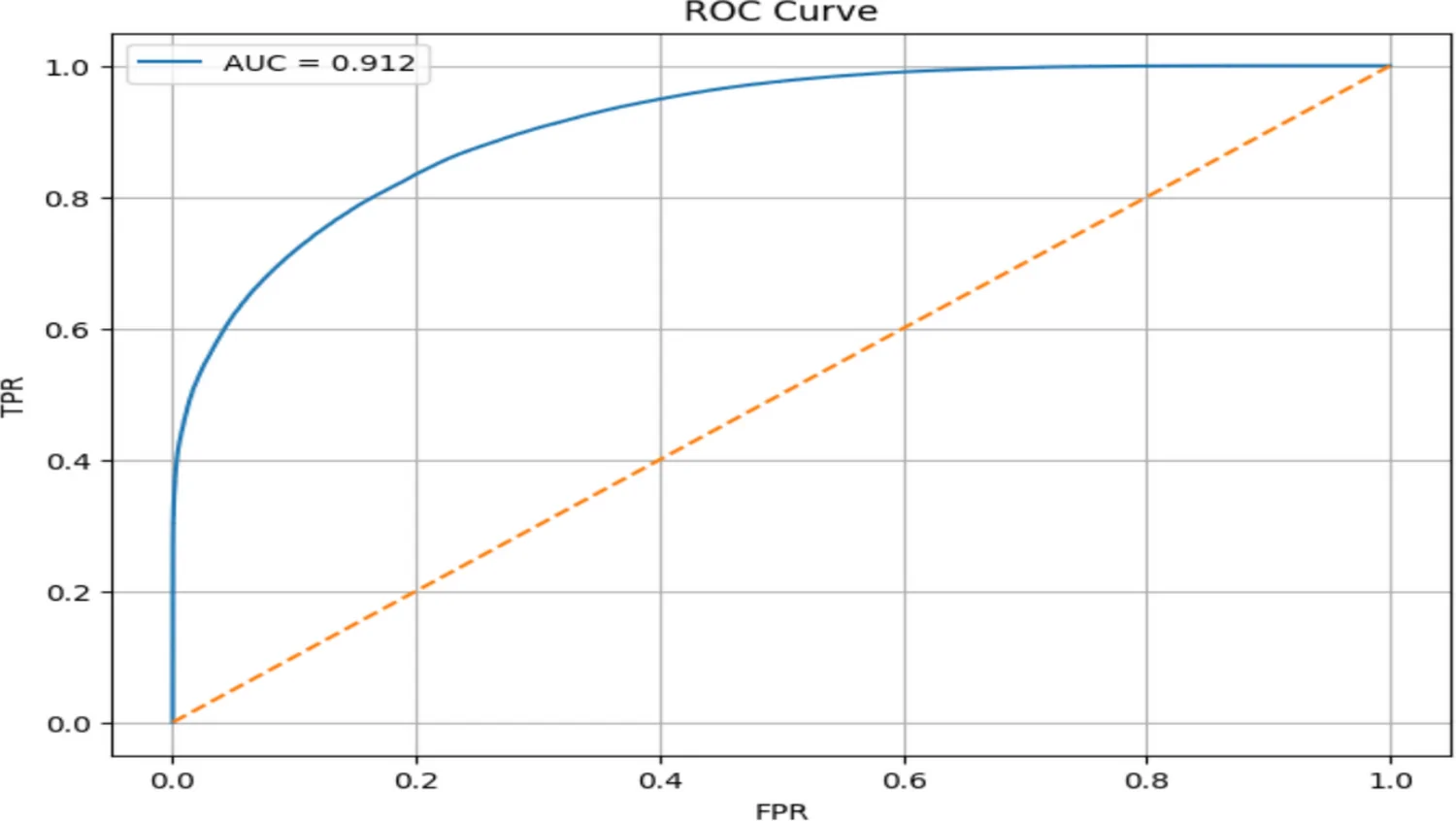
RAD-DINO ROC curve on the balanced test set, achieving an AUC of 0.912

A detailed breakdown of the model’s predictions is provided by the patient-level confusion matrix (Fig. 5), calculated at the optimal threshold of 0.464. The model correctly classified 995 out of 1022 cancer patients (True Positives) and 935 out of 962 non-cancer patients (True Negatives). The receiver operating characteristic (ROC) curve analysis resulted in a robust area under the curve (AUC) of 0.912, signifying excellent discriminative capability between the cancer and non-cancer classes (Fig. 6). While patient-level outcomes are the primary endpoint, a more granular analysis of the model’s performance on individual slices is provided in Appendix (Fig. 16).

### Embeddings and Statistical Analysis

To validate that the embeddings meaningfully captured disease-specific information, we performed statistical, interpretability, and retrieval analyses.

We conducted inferential statistical tests on the per-patient embedding vector norms to determine if a significant difference existed between the cancer and non-cancer cohorts. While an independent samples *T*-test was borderline (*t* = 1.934, *p* = 0.0533), a non-parametric Mann–Whitney *U* test confirmed a statistically significant difference in the embedding distributions (*U* = 522,292.500, *p* = 0.0160). This result provides quantitative evidence that the self-supervised model learned to encode clinically relevant, separable features for the two patient classes.

**Fig. 7:**
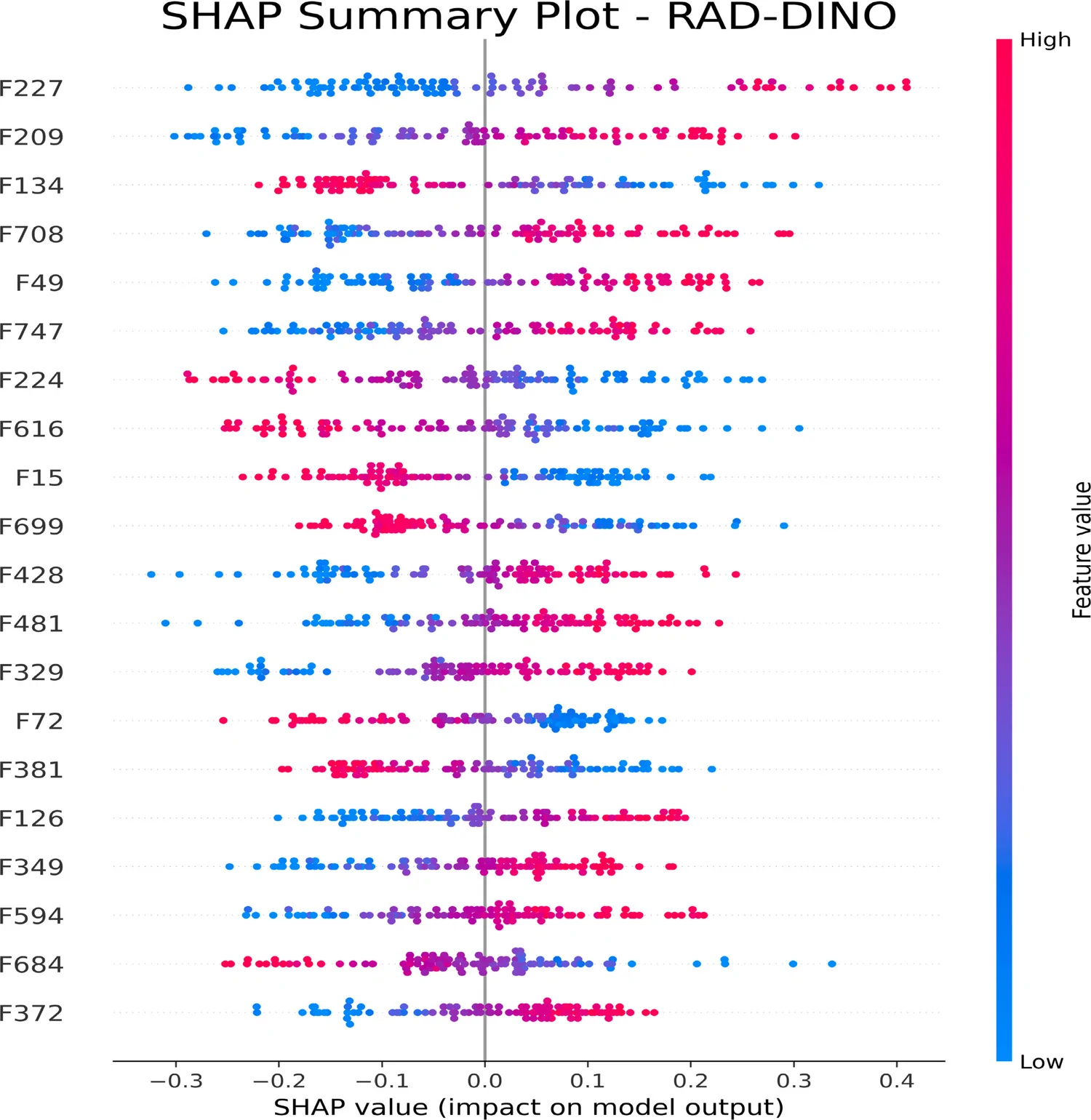
SHAP summary plot showing the impact of the top-20 features from the RAD-DINO embeddings on the model’s output

To provide insight into the model’s decision-making process, we employed SHAP (SHapley Additive exPlanations) analysis to identify which of the 768 features in the RAD-DINO embedding vector had the most significant impact on classification. The SHAP summary plot (Fig. 7) visualizes the contribution of the top-20 features. It reveals that high values for certain features (e.g., F227, F134) consistently pushed the prediction toward the cancer class, while others had varying effects. This analysis enhances model interpretability by pinpointing the key learned features that drive the diagnostic prediction and justify the classification AUC/ROC.

RAD-DINO’s latent features exhibit consistent, directional effects on the decision. The top-20 embedding dimensions by mean |SHAP| are shown in Appendix (Fig. 22A (bar) and Fig. 22B (beeswarm)), revealing features whose higher values reliably increase or decrease cancer risk. On the 60-patient SHAP set, the model’s top-2 probability slices had significantly larger irectional SHAP than the remaining slices (Wilcoxon* W* = 1267, *p* = 0.0048; effect size *r* = 0.335; median Δ = + 0.52 logit-SHAP, 95% CI 0.23–1.24). In contrast, an unpaired slice-level test was not significant (Mann–Whitney *U* = 11 593, *p* = 0.141; Cliff’s *δ* = 0.073), indicating a patient-specific concentration rather than a global shift. Appendix (Fig. 22C) summarizes top-2 vs. other slices; Appendix (Fig. 21) shows, per patient, how SHAP decreases with slice rank (more negative Spearman *ρ* indicates stronger concentration). Together with the rank-score traces Appendix (Figs. 19 and 20), these results support an intuitive workflow: triage by patient score, then inspect the highest-scoring slices.

Label-colored t-SNE plots provide qualitative views of the embedding spaces: RAD-DINO shows some class structure while CT-Foundation appears more diffuse (Figs. 14 and 15, Appendix). We avoid strong separability claims and emphasize quantitative metrics in imbalanced tests. This result highlights that RAD-DINO captures rich latent representations that go beyond binary classification. The learned features may correspond to underlying imaging phenotypes, such as nodule shape, size, lung texture patterns, or subtle anatomical variations. While a two-cluster separation would be expected for cancer prediction tasks, the emergence of multiple feature clusters suggests the potential for fine-grained stratification, which does assist downstream clinical subgroup discovery or radiomic biomarker exploration.

These interpretability analyses can assist radiologists in understanding model focus and verifying whether salient regions correspond to clinically relevant findings, thereby supporting more transparent and trust-aware AI decision support in screening workflows.

### Performance on Similar-Case Retrieval

After fine-tuning the embeddings with triplet loss to optimize for semantic similarity, the model was evaluated on the patient-level retrieval task. To quantify performance, we used Precision@5 (P@5)—the proportion of the 5 most similar retrieved patients sharing the correct diagnosis—a metric chosen for its clinical utility and robust evaluation. The fine-tuned embeddings achieved a Precision@5 of 0.853. This result demonstrates that the general-purpose features learned by RAD-DINO can be successfully adapted through further training to perform more complex semantic tasks beyond simple binary classification. The high precision in a retrieval context proves that the fine-tuning process did not merely learn a simple boundary between classes, but successfully organized the data into meaningful, coherent clusters. This confirms that the embeddings are robust enough for sophisticated applications like identifying patient subtypes or powering content-based diagnostic search engines.

### Model Training and Optimization

**Fig. 8:**
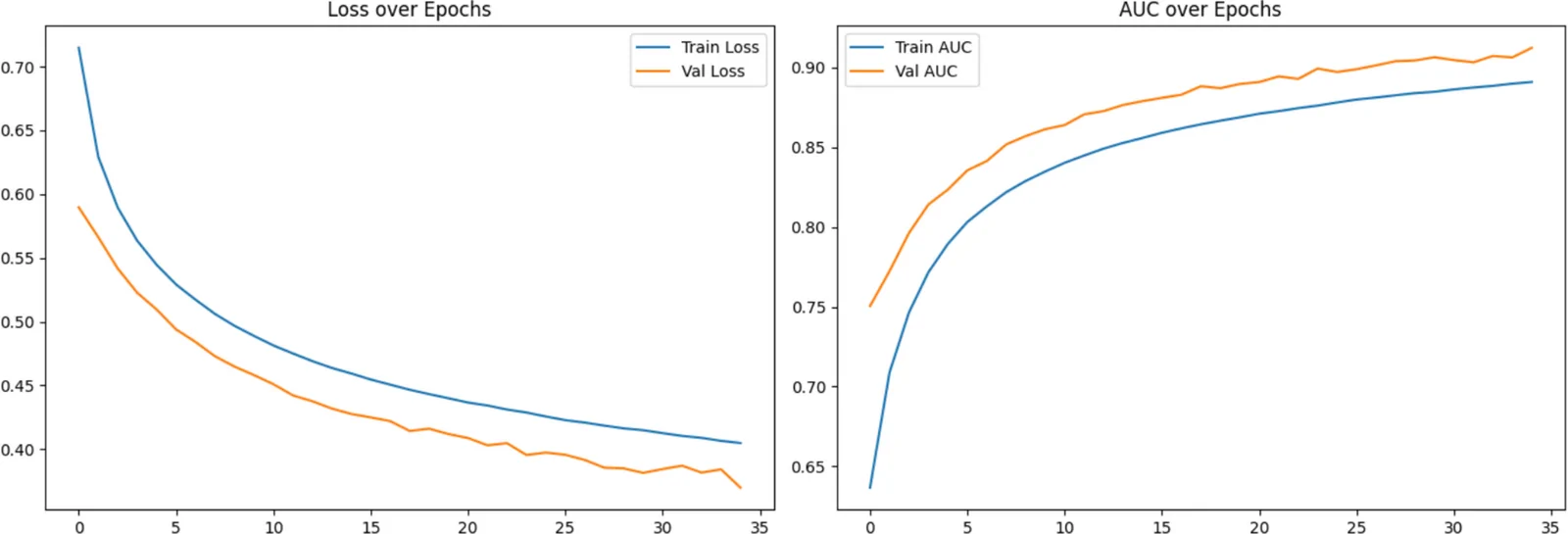
Training and validation curves (loss and AUC) over 35 epochs for the classifier trained on RAD-DINO embeddings

**Fig. 9:**
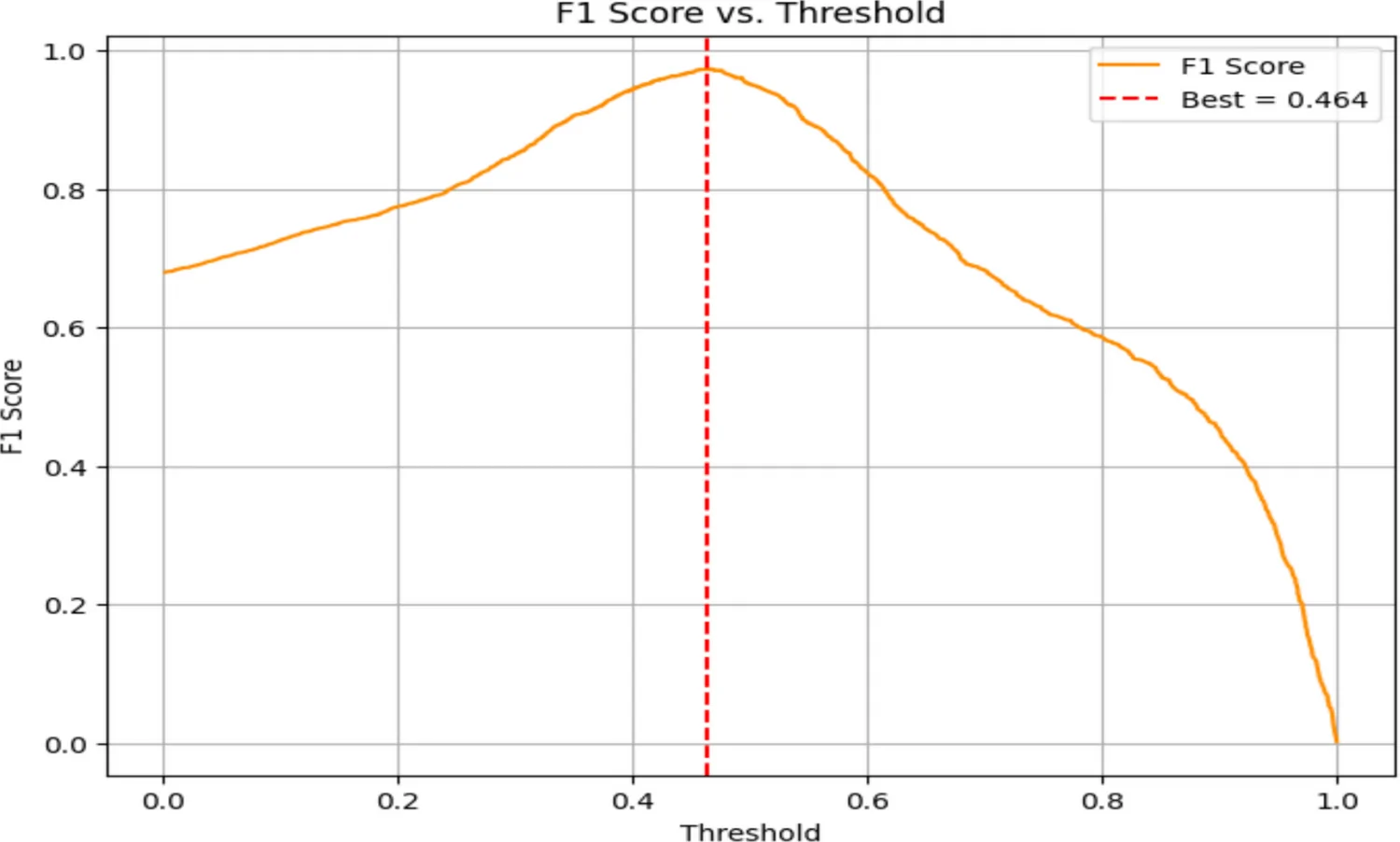
Threshold optimization curve illustrating the optimal balance between precision and recall for the RAD-DINO-based classifier at a threshold of 0.464

The training process for the downstream MLP classifier was stable and efficient. Training curves demonstrated smooth convergence, with the validation AUC reaching its plateau of 0.912 as the validation loss stabilized after approximately 20 epochs. This indicates effective generalization from the embeddings without signs of overfitting (Fig. 8). To maximize the F1-score, the optimal classification threshold was determined to be 0.464 based on the validation set performance (Fig. 9).

#### Apples-to-Apples 2D Baseline (ResNet-18) vs. RAD-DINO

On the same 60-patient test cohort (44/16, ~ 73% cancer), RAD-DINO substantially outperforms a supervised ResNet-18 trained on slices with patient-level aggregation. RAD-DINO achieves ROC-AUC = 0.960 (95% bootstrap CI: 0.91–0.99) and PR-AUC = 0.987 (0.97–1.00); after calibration, Brier = 0.099 and ECE@10 = 0.042. ResNet-18 reaches ROC-AUC = 0.715 (95% CI: 0.56–0.85) and PR-AUC = 0.850 (baseline precision = 0.733), with Brier = 0.183 and ECE@10 = 0.117. Thus, RAD-DINO improves ROC-AUC by + 0.245 and PR-AUC by + 0.137, while also showing better calibration (ΔBrier = − 0.084; ΔECE = − 0.075). At the test Max-F1 threshold, RAD-DINO (raw scores) attains precision = 0.98, recall = 0.93, and specificity = 0.94 (threshold ≈ 0.697; TP/FP/TN/FN = 41/1/15/3); with calibrated scores, precision = 0.89, recall = 0.95, and specificity = 0.69 (threshold ≈ 0.214; TP/FP/TN/FN = 42/5/11/2). These results indicate that—even without any CT-specific pretraining or fine-tuning—2D RAD-DINO features provide a stronger and better-calibrated signal than a supervised 2D CNN trained slice-wise under identical conditions (Table 3).

## Discussion

RAD-DINO’s architecture, based on native-resolution 2D axial CT slices, integrates naturally into radiologists’ diagnostic workflows. The model generates slice-level malignancy scores, enabling visualization of the most suspicious regions—a property that aligns with how clinicians interpret LDCT scans in practice. Although classification was ultimately aggregated at the patient level using mean aggregation, the model inherently provides per-slice predictions. Such granularity supports clinical transparency and could facilitate future applications in radiologist-AI collaboration or explainable CAD [9, 22].

By contrast, models like CT-Foundation [15] and Harvard’s CT-FM [23] operate on entire volumetric scans. CT-Foundation, for instance, generates a single 1408-dimensional embedding per volume via a video transformer pretrained on over 500,000 CTs paired with reports. Similarly, CT-FM employs cross-modal contrastive learning on large-scale paired image–text datasets to create highly generalizable representations across body regions. These models offer high scalability and robust pretraining, but often lack intrinsic spatial localization—making additional interpretability modules necessary for slice-wise review. While Stanford’s MERLIN [24] model enhances volumetric prediction by integrating radiomic and deep features, its complexity and dependence on 3D inputs limit generalizability in radiological-workflow settings.

To better understand how the models internally structure cancer-relevant features, we visualized embeddings via PCA and t-SNE. RAD-DINO’s embeddings exhibited tight clustering between cancer and non-cancer cases, achieving a silhouette score of 0.35. This indicates well-separated feature spaces and suggests strong task-specific encoding (see Appendix Fig. 14).

RAD-DINO offers practical advantages in terms of training and deployment. It was trained end-to-end on local hardware (2 GPUs) in under 8 h and supports real-time inference with minimal computational overhead. This contrasts with large foundation models [25] like CT-Foundation and CT-FM, which require extensive computation for pretraining and longer inference times, often via cloud APIs. As noted in prior reviews of scalable medical AI [26], lightweight models are particularly suited for rural clinics and point-of-care deployment where access to high-performance infrastructure may be limited.

While we aimed to include a direct comparison with CT-Foundation, differences in data preprocessing and input dimensionality prevented a strict side-by-side evaluation. CT-Foundation requires 3D volumetric inputs with isotropic resampling, whereas our pipeline was intentionally designed for native-resolution 2D slices without interpolation. Consequently, a direct quantitative comparison would confound model capability with preprocessing differences. We therefore report CT-Foundation metrics from its published NLST-based analysis for qualitative reference only. Readers should interpret this not as a head-to-head benchmark but as contextual evidence that 2D slice-based methods can achieve comparable performance at substantially lower computational cost.

**Fig. 10:**
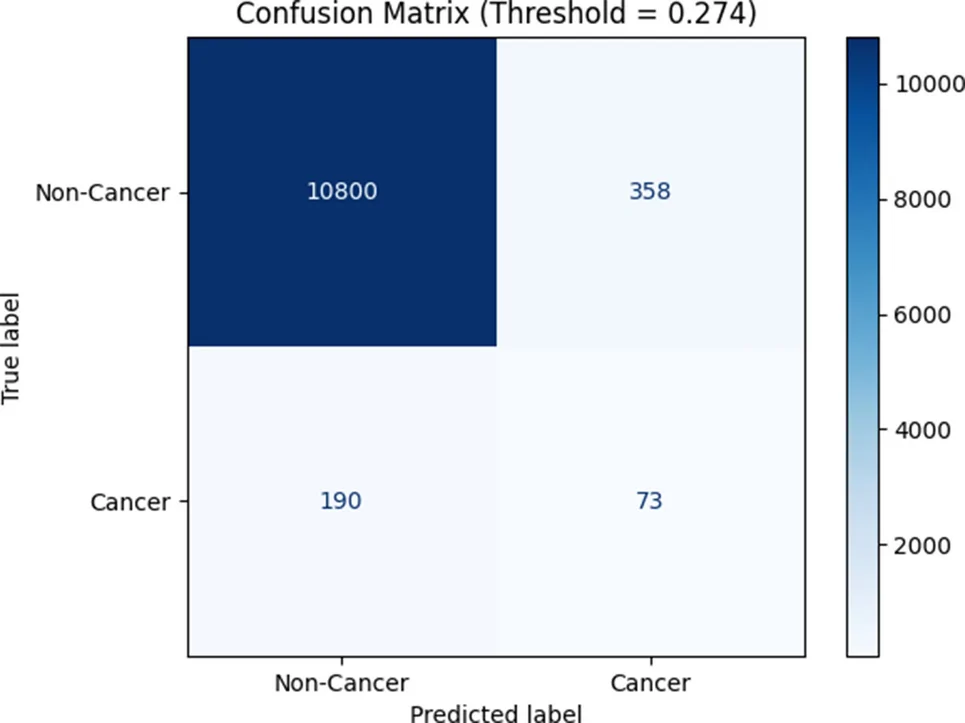
Confusion matrix for the CT-Foundation model evaluated on the imbalanced NLST test set at a threshold of 0.274

**Fig. 11:**
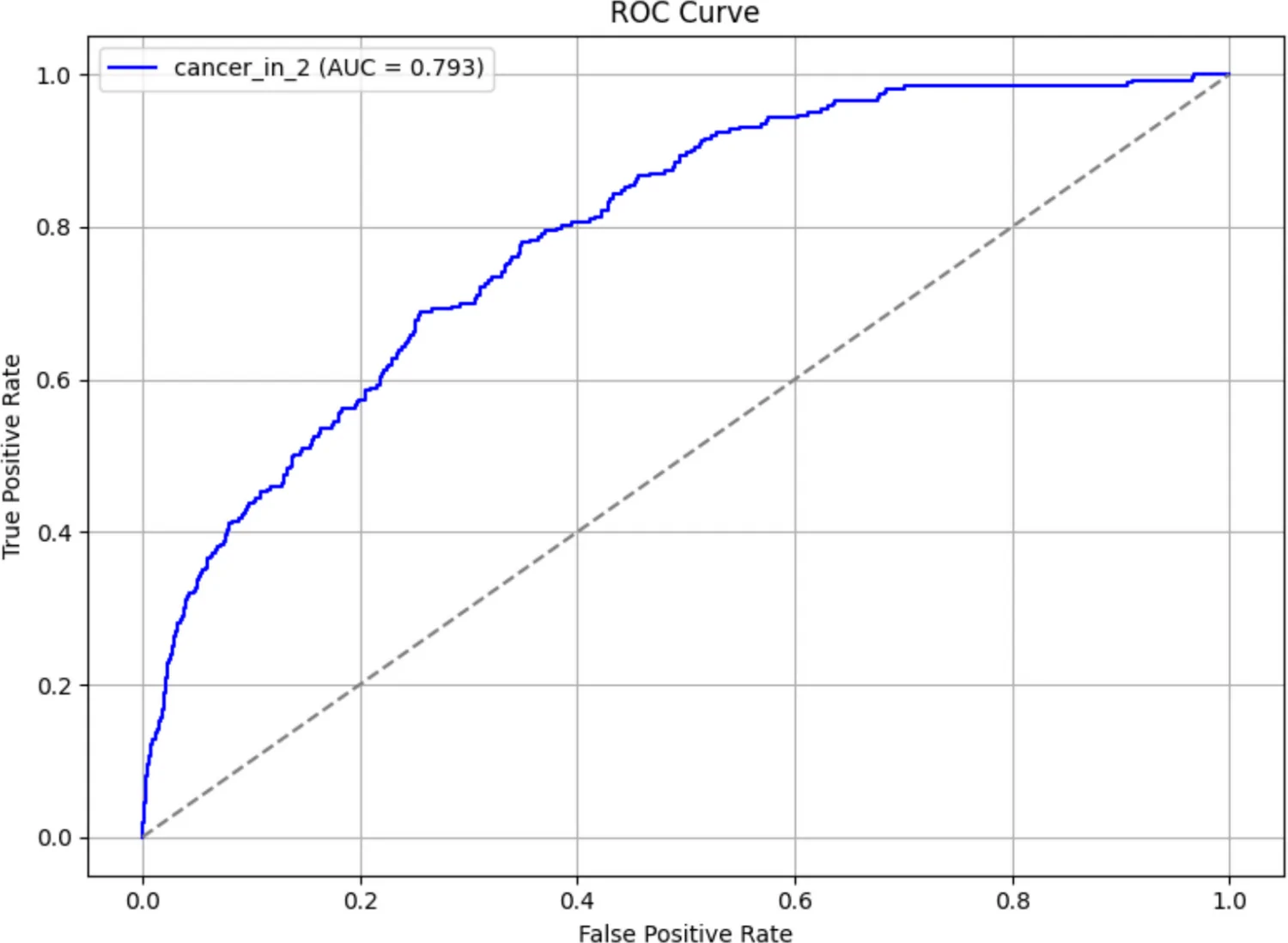
ROC curve for the CT-Foundation model, yielding an AUC of 0.793 on the imbalanced test set

**Fig. 12:**
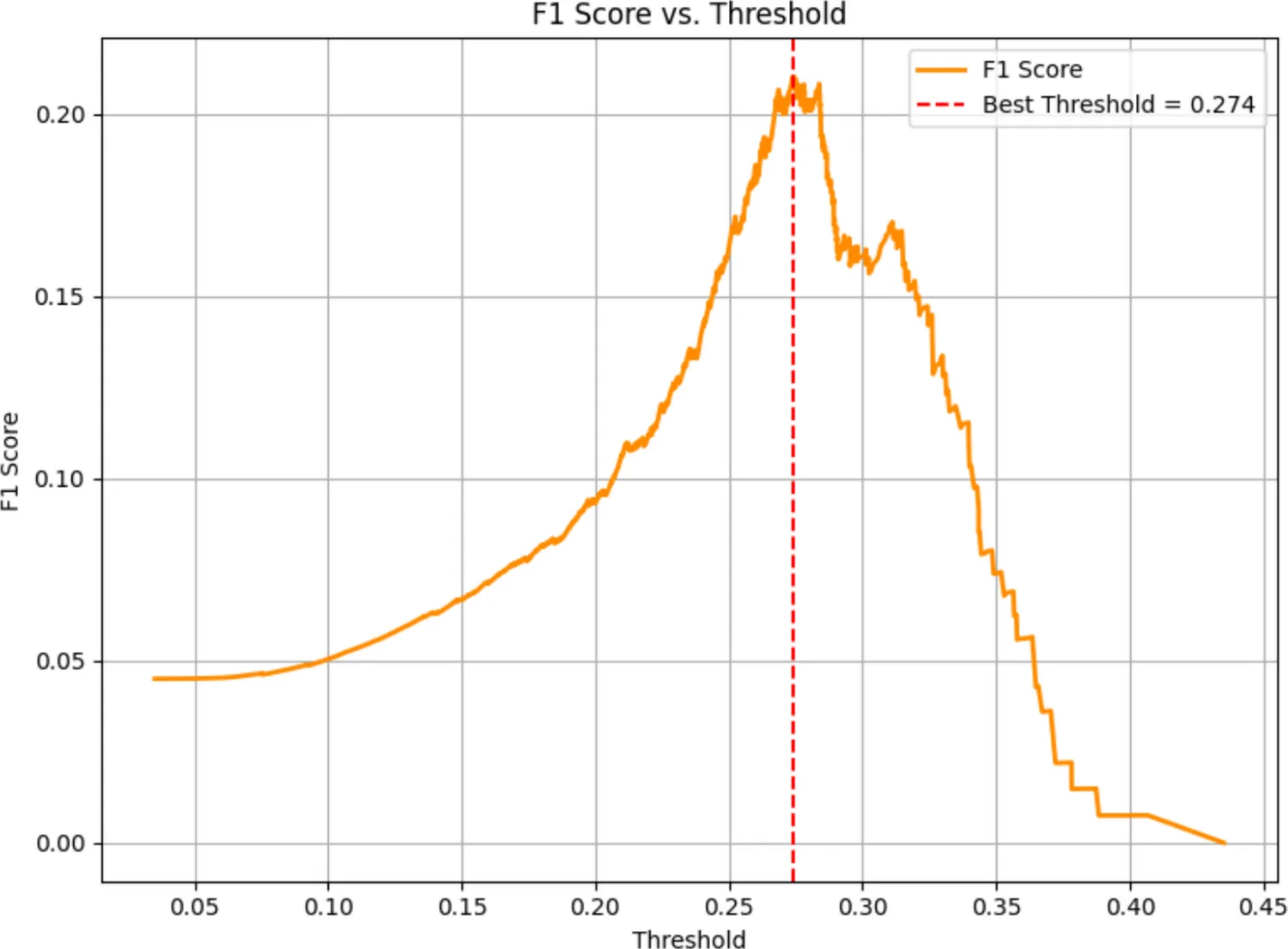
Threshold optimization curve for the CT-Foundation model, showing the optimal F1-score at a threshold of 0.274

**Fig. 13:**
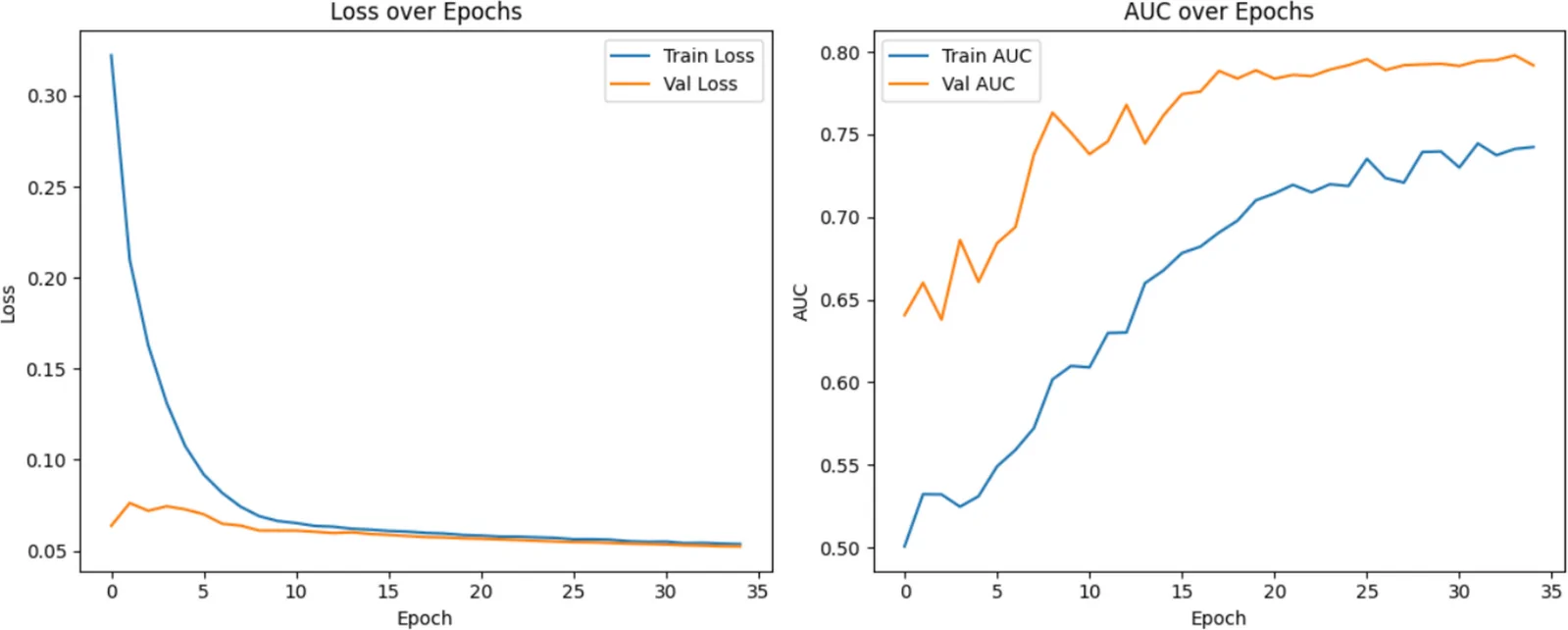
Training curves (loss and AUC) over 35 epochs for the classifier trained on CT-Foundation embeddings

**Fig. 14:**
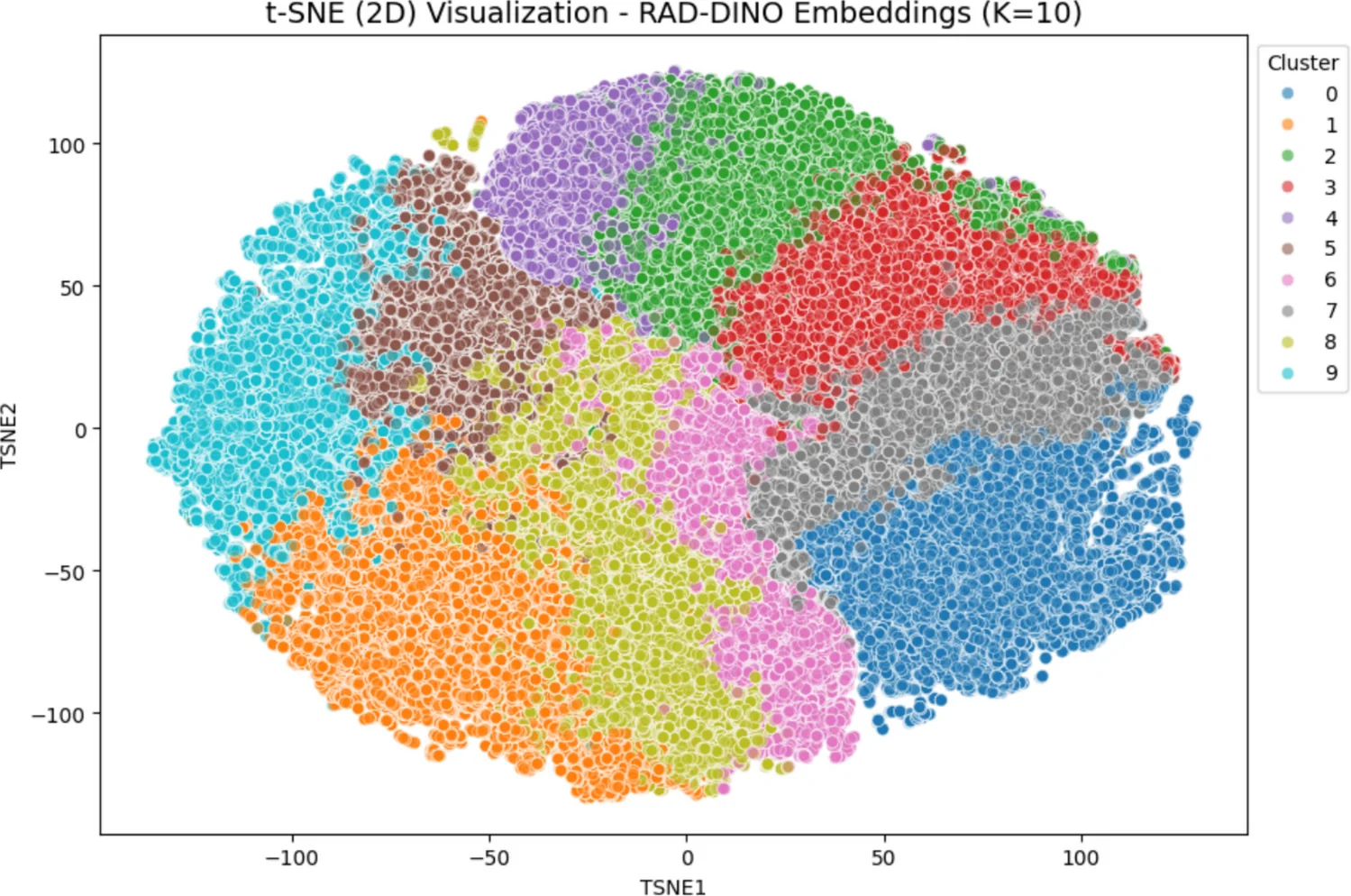
t-SNE visualization of the patient-level embeddings generated by the RAD-DINO model on the balanced cohort.

**Fig. 15:**
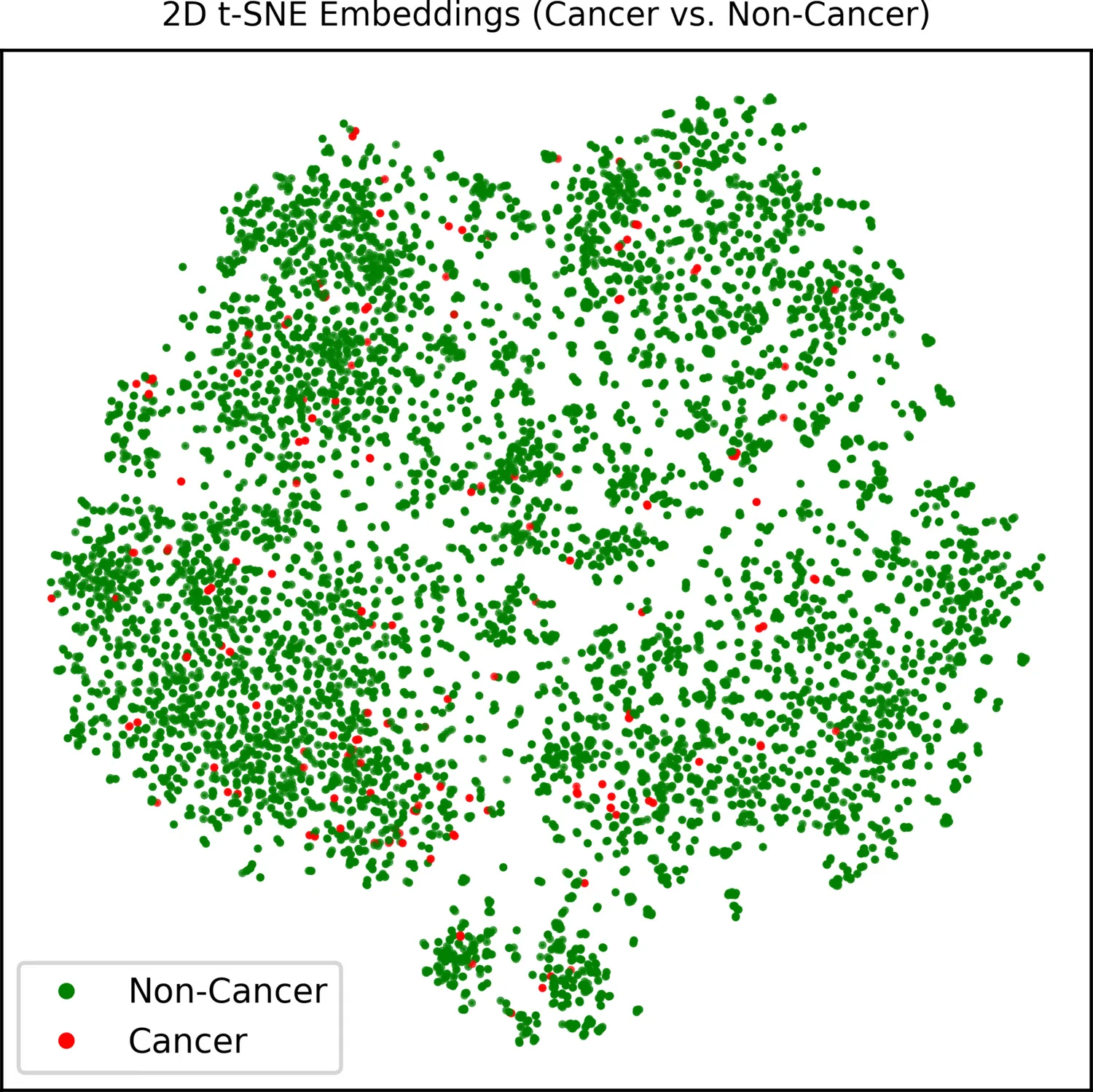
t-SNE visualization of the patient-level embeddings generated by the CT-Foundation model on the imbalanced cohort.

**Fig. 16:**
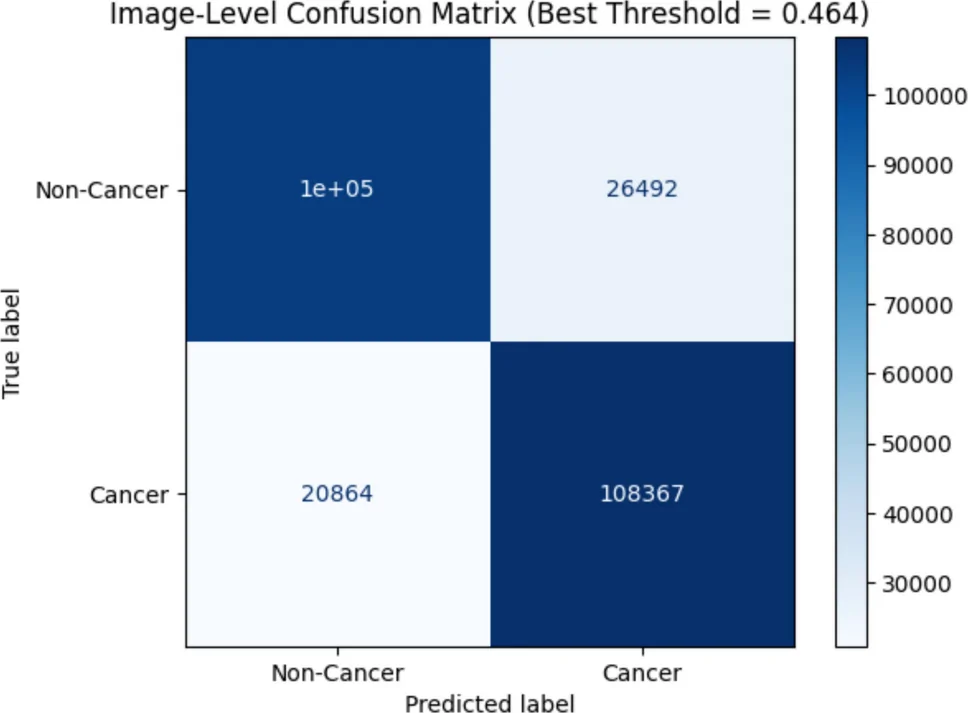
RAD-DINO slice-level (image-level) confusion matrix on the balanced test set, evaluated at a threshold of 0.464

**Fig. 19:**
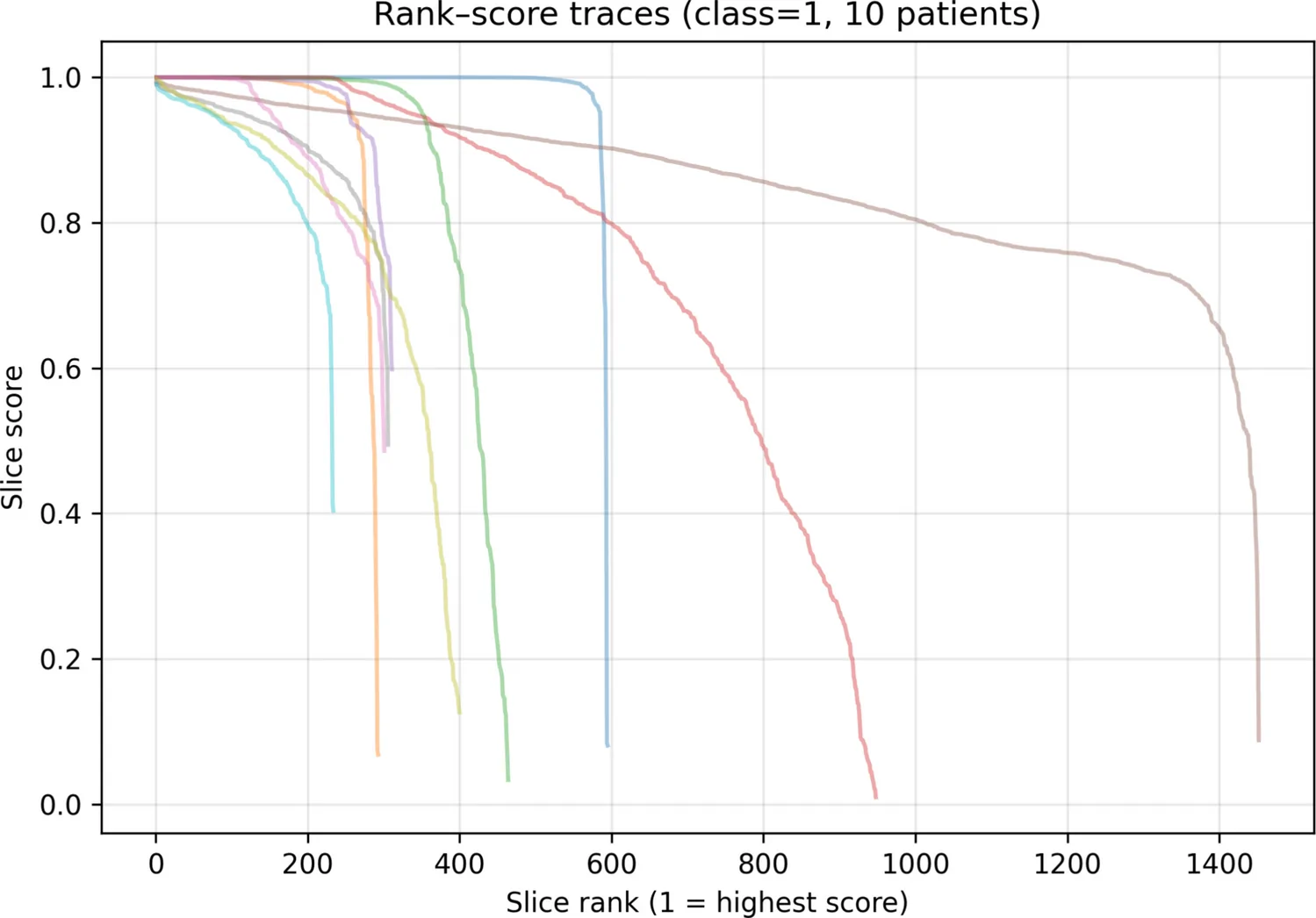
For 10 randomly selected positive patients, scores remain high across the top-ranked slices, indicating concentrated signal

**Fig. 20:**
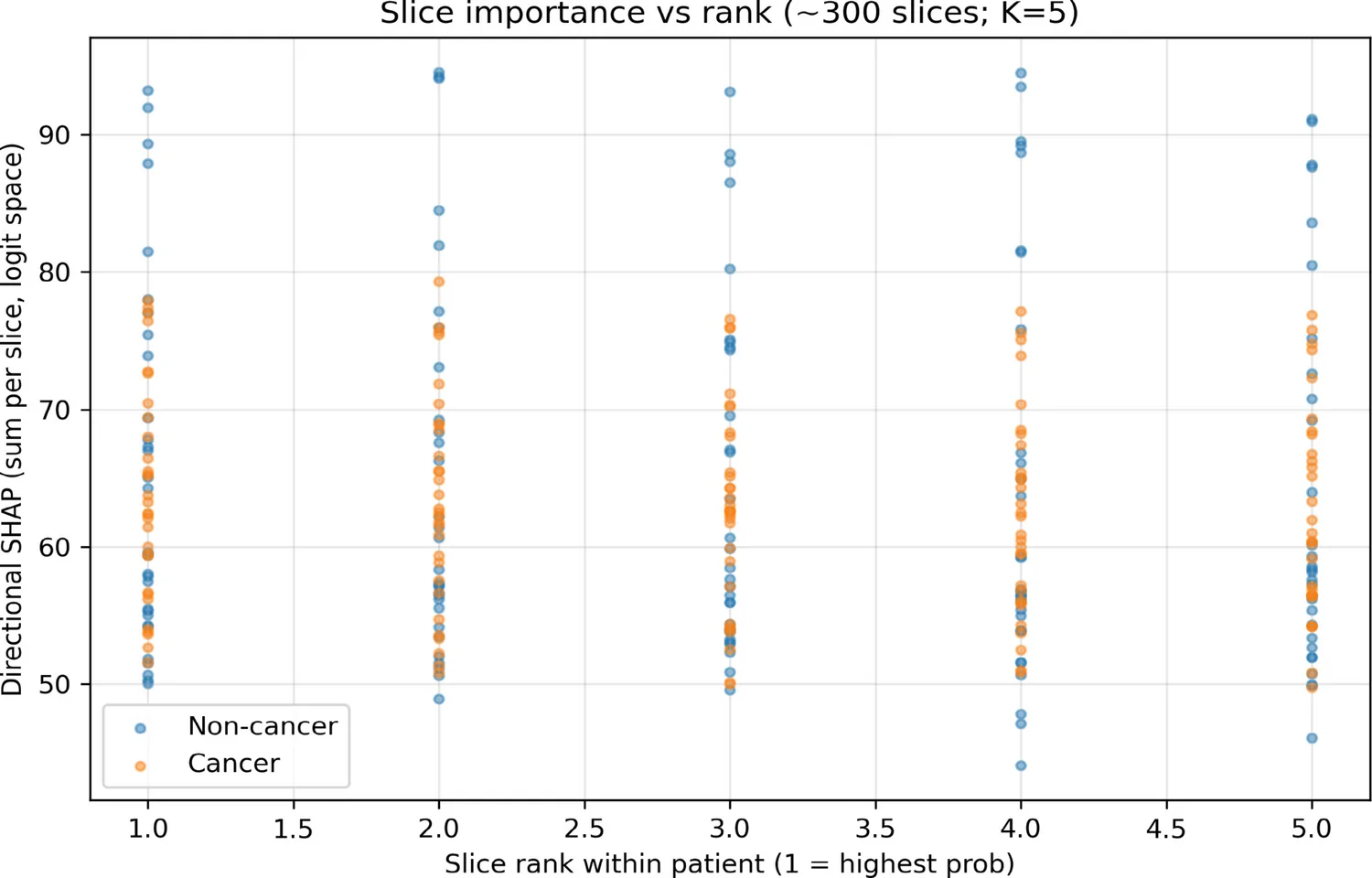
Directional SHAP (*y*-axis) vs. slice rank within patient; points jittered for visibility

**Fig. 21:**
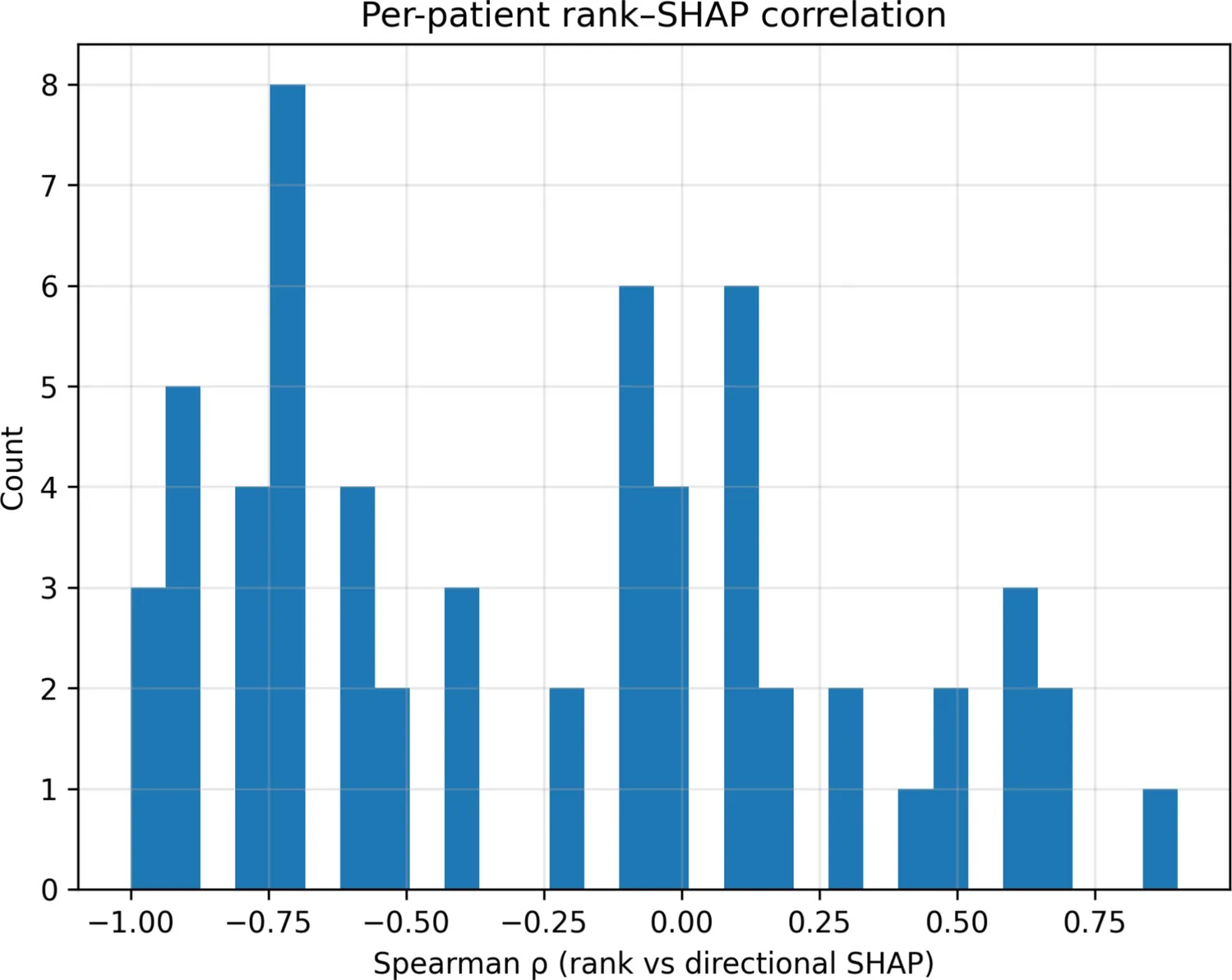
Histogram of per-patient Spearman *ρ* (rank vs. SHAP); more negative *ρ* indicates stronger within-patient concentration

**Fig. 22:**
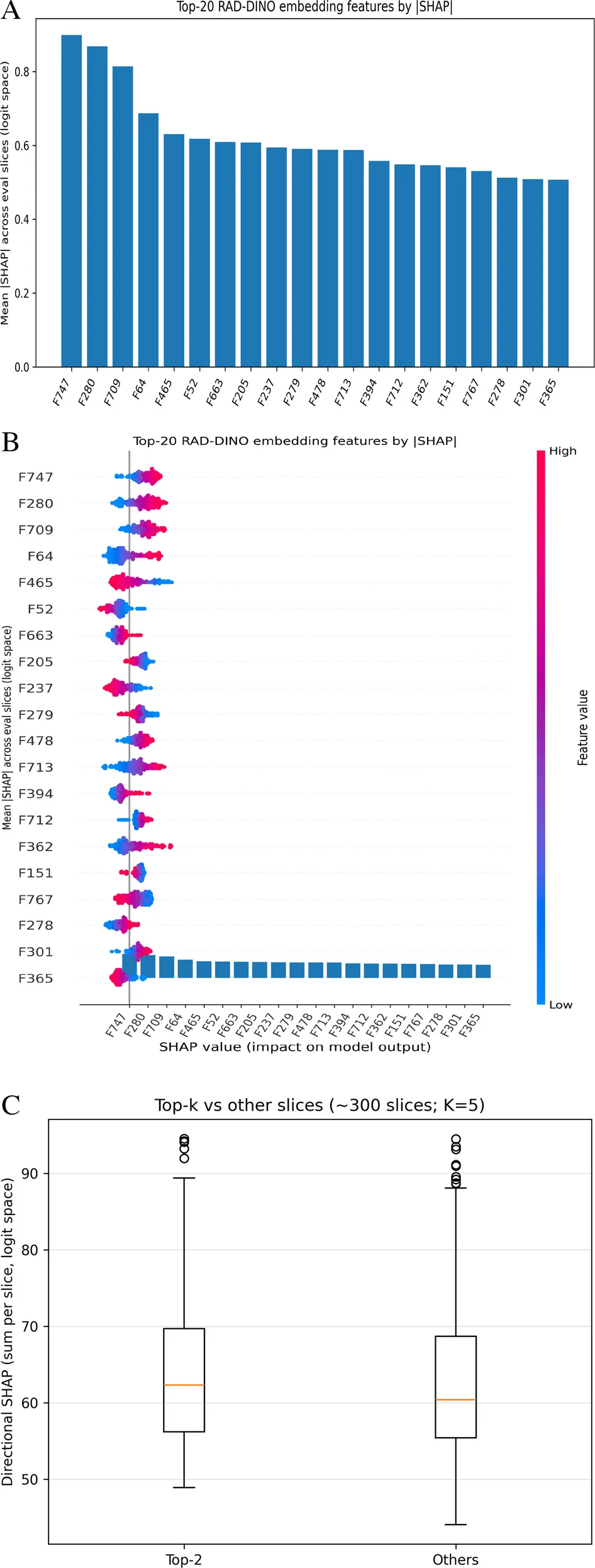
**A** Top-20 RAD-DINO embedding features by mean |SHAP|. Bar plot of mean absolute SHAP across the evaluation set highlights the most influential latent dimensions in the classifier’s logit space. **B** Beeswarm of SHAP for the top-20 features. Each point is a slice; color encodes feature value (low → high). Positive values push toward cancer; negative toward non-cancer. **C** SHAP concentration on high-probability slices. Within-patient directional SHAP is higher for the top-2 slices than the remaining top-5 slices (Wilcoxon *p* = 0.0048; median Δ = + 0.52 logit-SHAP). Boxes show IQR; whiskers show 1.5 × IQR

Our findings support a growing body of evidence that 2D slice-based models—especially those pretrained using self-supervision—can achieve competitive results in thoracic imaging tasks. By offering interpretability, computational efficiency, and clinically aligned outputs, models like RAD-DINO provide a feasible pathway for integrating AI into real-world lung cancer screening, particularly in rural and under-resourced settings. Future research may explore hybrid pipelines that combine volumetric embeddings (e.g., CT-FM, MERLIN) with slice-wise interpretability to deliver the best of both paradigms. Our study was designed to assess the foundational capability of the RAD-DINO model under controlled, class-balanced conditions and real-world class-imbalanced conditions. Future work should also include validation on external datasets from different institutions to ensure generalizability after extensive pretraining and fine-tuning of RAD-DINO. Finally, while we have established a strong performance baseline, direct comparisons to other architectures, such as volumetric models (see Appendix Figs. , 11, 12, 13, 14, and 15 and Tables 4 and 5), can provide a broader context for the trade-offs between different modeling strategies. As shown in Appendix (Figs. 10, 11, 12, 13, 14, and 15 and Tables and 5), we implemented the CT-Foundation model from Google Research under similar conditions, except for the balanced data cohort and the input image dimensions.

Our SHAP analysis suggests that RAD-DINO’s slice-level evidence is localized and directional: a small subset of high-probability slices carries most of the explanatory mass, and specific latent features consistently push predictions toward or away from cancer. While these embedding dimensions are not directly named radiomic traits, their effects can be visualized and linked to exemplar slices; we therefore frame them as *learned imaging patterns associated with malignancy risk*, not as hand-crafted features (additional SHAP diagnostics in Appendix).

Our main evaluation now reflects screening prevalence via repeated ≈6% draws with patient-level calibration/thresholds. A limitation is that, within NLST, we could not enrich the non-cancer pool for *positive screens* (e.g., Lung-RADS ≥ 3) [27] because the publicly released NLST tables we used do not include Lung-RADS annotations. Moreover, Lung-RADS constitutes an expert label; using it to curate training/evaluation sets would run counter to the label-efficient objective of this study (foundation embeddings + minimal supervision). We therefore report outcomes defined purely by the 2-year cancer window, and we make this trade-off explicit. In future work—contingent on access to Lung-RADS or an equivalent screening-decision proxy—we will provide sensitivity analyses restricted to Lung-RADS ≥ 3/4 and compare directly against radiologist triage endpoints (e.g., false-positive follow-ups).

RAD-DINO was pretrained on chest X-rays, not CT volumes or 2D scans, so a modality shift remains. We nevertheless observe transferable signal because self-supervised DINO/contrastive training tends to learn shape/edge/texture primitives and robust invariances that carry across projection and tomographic domains. That said, the shift likely limits the ceiling and affects probability calibration (we saw the usual ROC–calibration trade-off when applying isotonic). We expect in-domain SSL—e.g., continuing pretraining on unlabeled CT slices, or a mixed CXR/CT curriculum with a lightweight adapter—to improve both discrimination and calibration; we outline this as concrete future work.

Slice-based 2D pipelines give inherent localization and simpler deployment, whereas 3D models exploit volumetric continuity. A full head-to-head on the same cohort was not feasible for this revision; we include a 2D ResNet-18 baseline under identical conditions showing clear gains for RAD-DINO (Table 3). For transparency, our hardware was Apple M3 Pro (MPS; 36 GB unified memory), batch = 128 for feature extraction, with throughput **≈**15 slices/s (≈180 scans/hour at median 300 slices/scan). We will release code to reproduce these numbers.

**Table 3:** Head-to-head 2D baseline on the same 60-patient test set (44/16). ROC-AUC/PR-AUC are from patient-level scores; Brier/ECE use isotonic-calibrated probabilities

Model	Pretraining	ROC-AUC	PR-AUC	Brier (cal)	ECE@10 (cal)
RAD-DINO (frozen + MLP)	Radiology (2D SSL)	0.960	0.987	0.099	0.042
ResNet-18 (slice-trained)	ImageNet	0.715	0.850	0.183	0.117

This is a single-cohort study on NLST; although we report a clinically realistic evaluation at ≈6% prevalence using 25 repeated draws with patient-level splits, external validation across sites and scanners remains future work. Our labels reflect cancer within 0–24 months of the index scan, consistent with NLST screening follow-up horizons; we did not vary this window here, and a planned sensitivity analysis (e.g., 12- and 36-month windows) will assess robustness. We used one CT per patient and enforced patient-level splits to avoid leakage; nevertheless, we did not implement a volumetric (3D) baseline due to licensing/access constraints and to keep the study focused on a label-efficient 2D SSL pipeline. Finally, performance under real-world prevalence will depend on operational thresholds and downstream workflows; we therefore report PR-AUC, ROC-AUC, and reliability, plus validation-selected operating points, to make deployment trade-offs explicit.

This feasibility study was restricted to a single-cohort evaluation using the publicly available NLST dataset. Although this ensured reproducibility and controlled bias, it limits assessment of generalizability across scanners, populations, and institutions. Future work will focus on external validation using additional datasets such as LIDC-IDRI and PLCO to evaluate cross-domain robustness and population-level consistency.

## Conclusion

This work demonstrates that a label-efficient, slice-native pipeline built on frozen RAD-DINO embeddings can deliver clinically meaningful patient-level lung cancer risk estimates from LDCT without lesion annotations or volumetric modeling. Under a clinically realistic screening setup (≈6% prevalence), using patient-level aggregation and isotonic calibration, the approach shows consistent discrimination and markedly improved reliability (see Figs. 2 and 3), with stability assessed over repeated draws and summarized as medians with 95% percentile intervals (Table 2). Complementarily, a balanced-cohort analysis serves as an upper-bound sanity check and confirms that the same plug-and-play embeddings support downstream classifiers and retrieval with minimal adaptation.

Beyond aggregate metrics, the method offers interpretable behavior that maps to clinical reading. Cancer cases sustain higher top-k slice scores, and a small prefix of high-confidence slices contributes most of the patient-level risk (Figs. 4, 5, 6, and 7), a pattern reinforced by rank-score traces and slice-level SHAP analyses (Fig. S1–S4). These views support an intuitive workflow—triage by patient score, then review the highest-scoring slices—while preserving native in-plane resolution and avoiding interpolation artifacts common in coarse volumetric resampling.

We also provide apples-to-apples baselines under identical preprocessing, aggregation, splits, and calibration. RAD-DINO outperforms both a 2D ImageNet-pretrained ResNet-18 and a linear probe on the same embeddings (Table 2), indicating that the foundation representation—rather than the classifier head alone—drives the gains. Practically, the slice-based design scales linearly with stack length, runs on modest hardware, and integrates cleanly with PACS-style viewing, making it a plausible candidate for screening triage or worklist prioritization in resource-constrained settings.

Important limitations remain. Our study is single-cohort (NLST) and lacks Lung-RADS labels; consequently, we could not report analyses restricted to positive screens (e.g., Lung-RADS ≥ 3) or reader-centric endpoints. NLST’s label infrastructure also precludes lesion-level audits. In addition, RAD-DINO was pretrained on chest radiographs rather than CT; this modality shift likely caps calibration and ceiling performance, even though contrastive SSL appears to transfer useful structure. Finally, we did not execute licensed 3D foundation models on the same cohort; instead, we reported runnable and methodologically symmetric 2D baselines.

These constraints are tractable and define clear next steps: external validation across scanners and institutions; sensitivity analyses focused on positive-screen subsets when Lung-RADS is available; CT-native (or mixed CT/CXR) self-supervised continuation to improve calibration and robustness; and prospective studies linking patient-level scores to follow-up burden and time to diagnosis. Taken together, our results support 2D foundation embeddings as a strong, pragmatic starting point for LDCT screening workflows—one that preserves spatial fidelity, affords transparent slice-level reasoning, and offers a practical path toward scalable clinical deployment.

## Figures and Tables

**Table 6:**
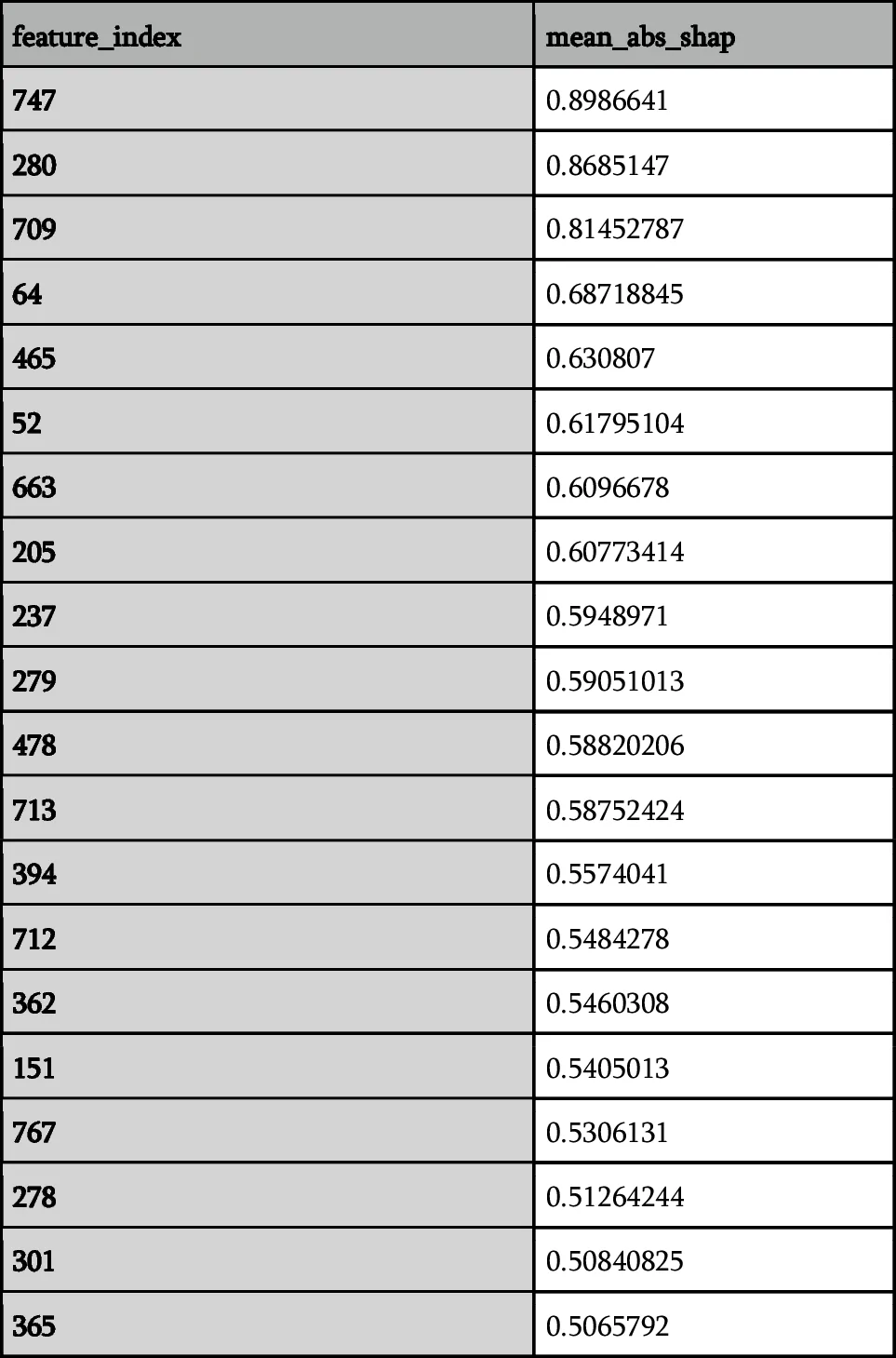
Top-20 features by mean |SHAP|

**Fig. 17:**
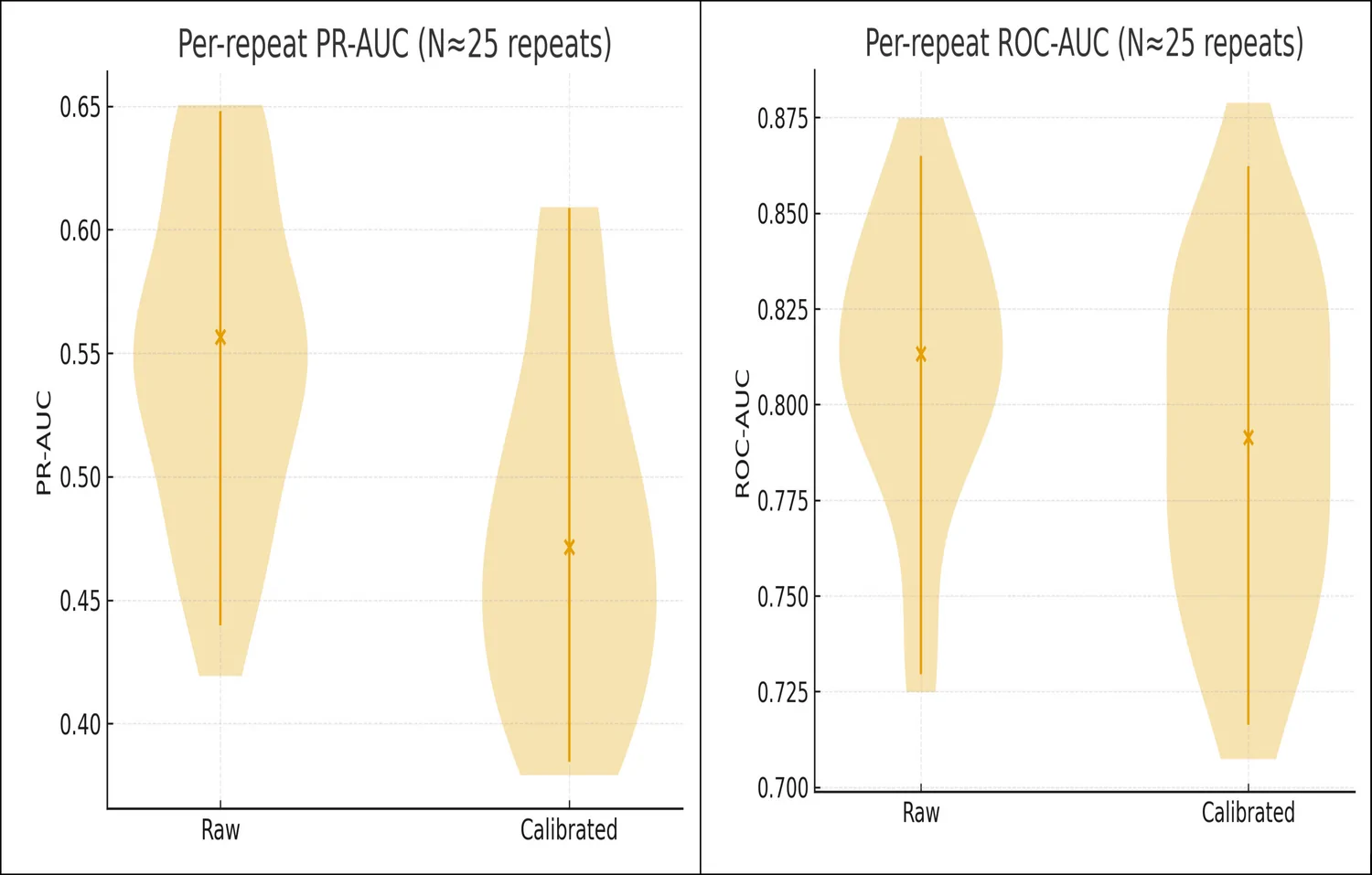
Per-repeat metrics across 25 imbalanced draws (≈6% prevalence). Violin plots show the distribution of **A** PR-AUC and **B** ROC-AUC for raw and calibrated patient-level predictions; dots mark the median and vertical bars indicate the 2.5th–97.5th percentile interval across repeats

**Fig. 18:**
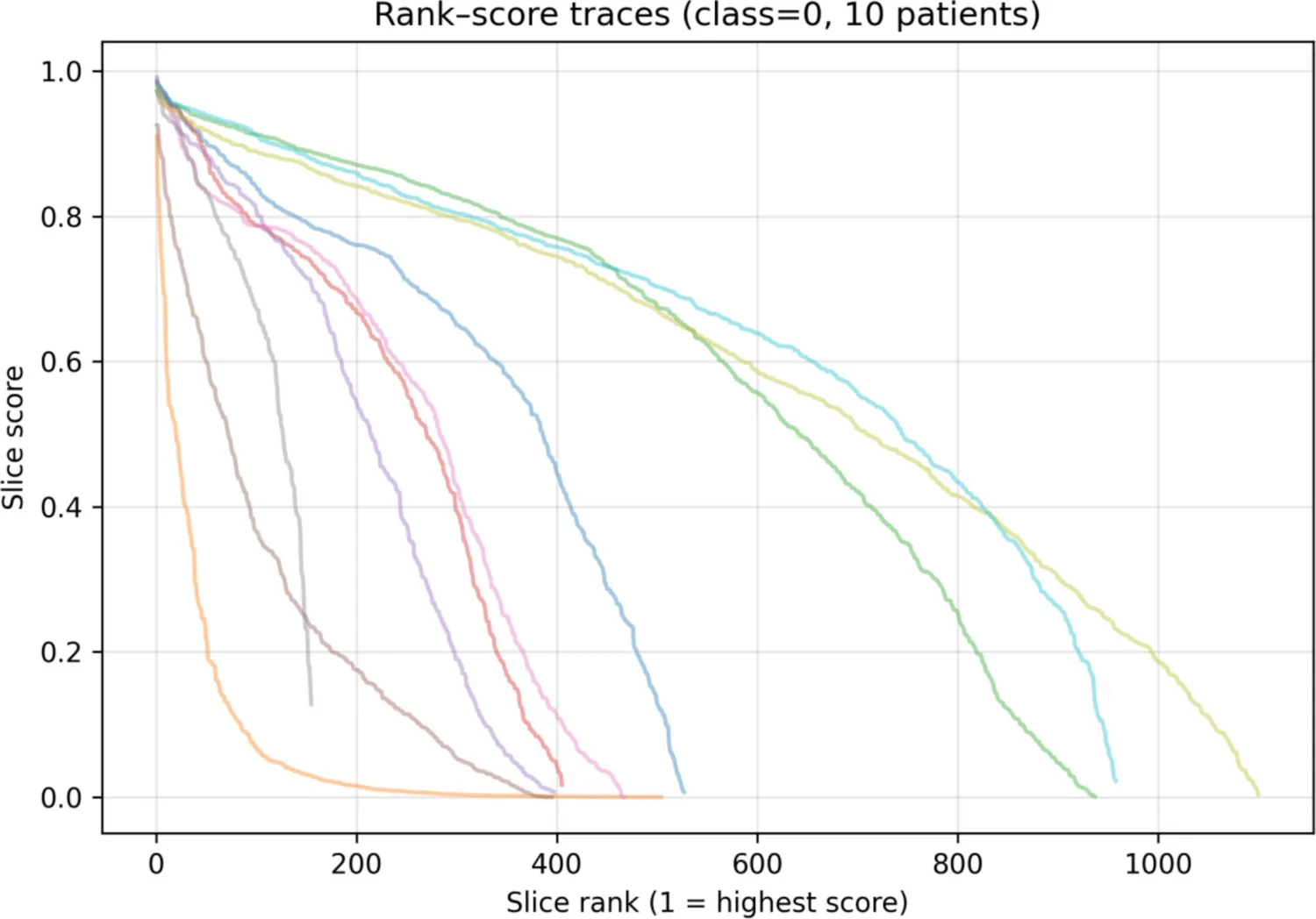
For 10 randomly selected negative patients, scores as a function of slice rank (1 = highest). Most negatives drop off quickly, indicating low sustained confidence

## Data Availability

The lung CT imaging data used in this study are publicly available through The Cancer Imaging Archive (TCIA) under the National Lung Screening Trial (NLST) collection. The dataset can be accessed at: https://www.cancerimagingarchive.net/collection/nlst/ All custom code used for preprocessing, model training, evaluation, and analysis will be made publicly available upon publication in a dedicated GitHub repository. A link to the repository will be added once the code is finalized and uploaded.

## Appendix

Contextual Analysis of the Volumetric CT-Foundation Model.

The following data is provided for contextual reference only. The results were generated using the publicly available CT-Foundation model on a naturally imbalanced cohort from the National Lung Screening Trial (NLST). As stated in the main text, these results are not intended for a direct, head-to-head performance comparison with the RAD-DINO model due to significant differences in model architecture (3D vs. 2D), input data processing, and evaluation cohort composition (imbalanced vs. balanced).

## Methods

The CT-Foundation model is a large-scale, 3D foundation model developed by Google Research. For this analysis, we used its publicly available API to extract pre-computed, 1408-dimensional feature embeddings for each patient’s full CT volume. As described by the model’s authors, the API internally handles the volumetric data, including resampling.

The evaluation was performed on a naturally imbalanced subset of the NLST dataset, representing a real-world screening distribution with a cancer prevalence of approximately 4–6%. A downstream multilayer perceptron (MLP) with a single hidden layer was trained on these embeddings for the classification task. To account for the significant class imbalance, a weighted binary cross-entropy loss function was used during training. For the retrieval task, the raw, pre-computed embeddings were used directly without any fine-tuning.

## Results

The performance of the CT-Foundation pipeline is detailed below.

Task 1: Patient-Level Cancer Classification—on the imbalanced test set, the model achieved a high accuracy primarily due to the large number of true negative cases. The key performance metrics are summarized in Table 2. The model’s confusion matrix, ROC curve, training dynamics, and threshold optimization are shown in Figs. 10, 11, 12, and 13. The optimal classification threshold was found to be 0.274.

**Table 4:** Patient-level classification performance of the CT-Foundation model on an imbalanced test set.

Model	Test set	Accuracy	Precision	Recall	F1-score	AUC
CT-Foundation	Imbalanced	0.937	0.271	0.277	0.274	0.793

Task 2: Patient-level similar-case retrieval—on the retrieval task, the CT-Foundation embeddings, used without any modification, achieved the performance shown in Table 3.

**Table 5:** Patient-Level Similar-Case Retrieval Performance for CT-Foundation

Model	Finet -Tuning	Precision@5
CT-Foundation	No (Used as is)	0.977

## Visualization of Embedding Spaces

To qualitatively assess the structure of the learned feature spaces, we used t-SNE to visualize the patient-level embeddings from both the RAD-DINO and CT-Foundation models. As noted in the main text, the RAD-DINO embeddings formed well-separated clusters (silhouette score = 0.35). In contrast, the CT-Foundation embeddings showed more diffuse class boundaries (silhouette score = 0.12). Figures 14 and 15 depict the t-SNE visualization of RAD-DINO and CT-Foundation.

## Slice-Level Classification Performance for RAD-DINO

To supplement the patient-level analysis presented in the main text, this section provides the classification performance at the individual slice (image) level. While patient-level outcomes are the most clinically relevant, slice-level performance demonstrates the model’s ability to accurately identify features within specific images.

The slice-level confusion matrix was generated from the balanced test set at the same optimal threshold of 0.464 used for the patient-level evaluation. As shown in Fig. 16, the model accurately identified a high number of cancer-positive slices while maintaining a low false-positive rate across all slices evaluated (Fig. 17). This strong slice-level performance is the foundation for the robust patient-level aggregation results (Table 6).
